# Evaluating the quality of systematic reviews and meta-analyses published in behaviour analysis journals: An umbrella review

**DOI:** 10.1371/journal.pone.0350142

**Published:** 2026-06-26

**Authors:** Richard May, Olivia Campbell, Magda Apanasionok, Josh Molina, Christopher Seel, Vaso Totsika, Aoife McTiernan, Alan Tennyson, Corinna Grindle, Simon Dymond

**Affiliations:** 1 School of Psychology and Therapeutic Studies, University of South Wales, Cardiff, United Kingdom; 2 The Centre for Research in Intellectual and Developmental Disabilities, University of Warwick, Coventry, United Kingdom; 3 Division of Psychiatry, University College London, London, United Kingdom; 4 University of Galway, Galway, Ireland; 5 Intellectual Disabilities Research Institute (IDRIS), University of Birmingham, Birmingham, United Kingdom; 6 School of Psychology, Swansea University, Swansea, United Kingdom; 7 Department of Psychology, Reykjavík University, Reykjavík, Iceland; Athens Medical Group, Psychiko Clinic, GREECE

## Abstract

High-quality systematic reviews and meta-analyses are essential for translating evidence into practice. We conducted an umbrella review to evaluate the methodological quality of systematic reviews and meta-analyses published in the field of behaviour analysis up to and including 2023. Eligible studies were identified through targeted searches of seven behaviour analysis journals using APA PsycINFO. Quality was assessed using the Assessing the Methodological Quality of Systematic Reviews (AMSTAR 2) and Revised AMSTAR (R-AMSTAR) instruments. Temporal trends were analysed using Bayesian multilevel regression. The protocol was preregistered on the Open Science Framework (doi.org/10.17605/OSF.IO/U38Z4). We identified 64 reviews (16 of which included a meta-analysis), all of which were rated as ‘critically low’ quality using the AMSTAR 2 criteria. Methodological shortcomings included absent protocol registration, inadequate risk of bias assessment, and failure to assess publication bias. Mean R-AMSTAR adherence was 43.8% (range 11–66%) and increased by 1.08% per year (population-level average marginal effect from the multilevel logistic model (95% CI [0.59, 1.58]), indicating robust methodological improvement over time. Currently, many systematic reviews and meta-analyses in behaviour analysis do not yet meet contemporary standards of methodological rigour. Enhancing the quality, transparency, and consistency of evidence synthesis is vital if systematic reviews are to meaningfully inform practice.

Systematic reviews (SR) and meta-analyses (MA) provide objective assessments of the depth and quality of research in a domain, helping to reveal gaps in understanding as well as orient future studies. They also play an important role in helping to communicate current knowledge in a way that can be easily understood by researchers, practitioners and policymakers [1,2,3]. It has been argued that SRs and MAs sit at the top of the so-called evidence pyramid, because they integrate the relevant evidence in a domain and thus provide information about the extent to which an intervention can be considered ‘evidence-based’ [4]. Given their status and potential to inform practice and policy, it is important that SRs and MAs are undertaken rigorously and reported in accordance with best practice guidelines.

In the field of behaviour analysis, SRs and MAs have the potential to play an important role in making sure that practice guidelines and clinical decision-making are based on the latest and best available evidence. Behaviour analysis is a branch of psychology grounded in the experimental study of learning, with a particular emphasis on how environmental contingencies shape behaviour [5,6,7]. Rooted in the principles of operant conditioning [8], it provides both a conceptual framework and a set of empirically validated methods for understanding and influencing behaviour. Over the past six decades, behaviour analysis has underpinned the development, delivery, and evaluation of interventions in many domains including education and special education, services for autistic people and those with intellectual and developmental disabilities, behavioural medicine (e.g., contingency management for substance use), and organisational behaviour management in workplaces [9]. Similar to developments in other applied psychological fields such as clinical and health psychology, behaviour analysis increasingly relies on systematic methods to synthesise findings and guide evidence-based practice. Systematic reviews hold particular importance for behaviour analysts for two reasons. First, behaviour analysts emphasise the link between evidence and practice [10], and SRs/MAs capture the state of the intervention evidence base, enabling practitioners to make evidence-informed clinical decisions. Second, behaviour analysis relies heavily on internal replication of experimental effects across small numbers of participants, typically through single-case experimental designs [11,12]. Given this approach, establishing generality (i.e., external validity) requires that studies be synthesised and appraised in a systematic and transparent manner [13]. The resulting literature is large and heterogeneous in which effects often vary by person, context, and implementation. Arguably, such conditions are precisely those under which systematic reviews and meta-analyses add the most value.

The number of systematic reviews and meta-analyses published in academic journals has increased markedly in recent years [14,15], and behaviour analysis is no exception. Examining 28 behaviour-analytic journals, King et al. [16] found that the publication of SRs and MAs has grown steadily over the past thirty years. Between 2015-2020, SRs and MAs represented more than half of all published review papers in their sample, having replaced narrative (literature) reviews as the most common review type. While the trend in formalised evidence synthesis is encouraging, we currently know very little about the extent to which the growing numbers of SRs and MAs in behaviour analysis adhere to current best practice in terms of methodology and reporting. This represents an important gap, as SRs of low quality risk misleading consumers of the research literature [5].

Outside of behaviour analysis, the growth of SRs and MAs has prompted best practice guidelines aimed at increasing rigour, transparency, and reproducibility. For example, the Preferred Reporting Items for Systematic Reviews and Meta-Analyses guidelines (PRISMA) [17,4] were developed to provide a standardised framework for authors to report their methods and findings. The latest version of the guideline consists of a 27-item checklist that specifies a minimum set of items for reporting, and is widely used in medicine, health and educational interventions. Whereas PRISMA is concerned with reporting quality, the AMSTAR 2 (Assessing the Methodological Quality of Systematic Reviews - 2nd Edition) [18] is a 16-item checklist designed to evaluate reporting *and* methodological characteristics of SRs/MAs of both randomised and non-randomised studies. The Revised-AMSTAR (R-AMSTAR) [19] is a version of the original AMSTAR instrument that has been adapted to more sensitively quantify the extent to which a SR/MA meets each criterion.

Both the AMSTAR 2 and R-AMSTAR have been used to appraise SRs and MAs across a range of disciplines [20–23]. For example, Matthias et al. [22] used the AMSTAR 2 to evaluate published SRs of treatments for adult major depression. The authors reported that the quality of the SR literature was overwhelmingly poor: 53 of the 60 included reviews received a “critically low” confidence rating, and fewer than 20% provided either a justification for included study designs or a full list of excluded studies. They concluded that, given these shortcomings, the existing systematic review literature may not be entirely reliable as a source of clinical guidance. Similarly, Jamshidi et al. [21] investigated the methodological quality of published meta-analyses involving single case experimental designs (SCEDs). While the authors detected improvements in methodological quality across time, more than 90% of eligible studies did not even reach a mid-point score (22 on a scale of 0-44) on the R-AMSTAR, suggesting significant room for improvement. Quality appraisal work has also been undertaken in closely related fields; for example, Maggin et al. [24] recently examined quality indicators for SRs in the behavioural disorders literature. To date, however, no published study has formally appraised the methodological quality of SRs and MAs within behaviour analysis as a discipline. Although Jamshidi et al., focused on SCEDs as a methodological category, neither this nor appraisal work in adjacent fields, speaks directly to the behaviour analysis literature.

In summary, SRs and MAs are intended to provide a comprehensive assessment of evidence in a way that minimises bias. In behaviour analysis, SRs are increasingly used for intervention selection and clinical decision making [16]. The absence of any field-specific quality appraisal, combined with the growing prevalence of SRs and MAs in behaviour analysis journals, makes this an opportune moment to evaluate the methodological standards of this literature. The purpose of the current study therefore was twofold. First, we sought to critically evaluate the characteristics and quality of SR/MAs published in behaviour analysis journals as measured by the AMSTAR 2 and R-AMSTAR. Second, we aimed to examine whether adherence to methodological and reporting standards identified by these tools has improved over time.

## Method

### Protocol and registration

The review protocol and statistical analysis plan was preregistered and is available on the Open Science Framework at the following link: https://doi.org/10.17605/OSF.IO/U38Z4. The review protocol was updated from a previously registered protocol. All the protocol revisions are documented in the supporting information.

### Eligibility criteria

Studies were included if they met the following inclusion criteria:

- Published in one of the following behaviour analysis journals: *Analysis of Verbal Behavior, Behavior Analysis in Practice, Education and Treatment of Children, Journal of Applied Behavior Analysis, Journal of the Experimental Analysis of Behavior, The Behavior Analyst, Perspectives on Behavior Science,* and *The Psychological Record*.
- Were systematic reviews or meta-analyses or combined systematic review and meta-analyses.
- Contained the phrase “systematic review” or “meta-analysis”/ “meta-analytic” or some variant thereof, in either the title or abstract of the study.

Studies were excluded if they were described as a critical review, narrative review, literature review, scoping review, or were a primary study reporting original data. There were no restrictions on the condition, domain, assessment, intervention or exposure being studied, or language of publication; however, we excluded reviews in which the focus was on non-human animal studies. No restrictions on publication date were specified.

In total, seven journals (*Perspectives on Behavior Science* was, up until 2018, titled *The Behavior Analyst*) were selected for inclusion as they represent all publications by the Association for Behavior Analysis International (ABAI) and the Society for the Experimental Analysis of Behavior (SEAB), with the exception of *Behavior and Social Issues*. We selected these publications for review as ABAI and SEAB are two of the most prominent professional membership organizations in behaviour analysis and therefore might be said to represent flagship behaviour analytic publications [16]. In addition, these journals are made available to those that are Board Certified Behavior Analysts (BCBAs), and members of the ABAI and are therefore likely to be key sources that practicing behaviour analysts use to support evidence-based decision making. *Behavior and Social Issues* was excluded because its primary focus on cultural systems analysis falls outside the scope of the intervention-focused literature appraised in the present review. Finally, we conducted a scoping exercise using the planned search protocol to estimate the likely yield of systematic reviews and meta-analyses. The projected volume was compatible with our screening and extraction capacity, so we adopted this set of journals as the sampling frame for the present study. The included journals had impact factors ranging from 0.8 to 3.8: *Journal of Applied Behavior Analysis (JABA; IF = 3.0), Perspectives on Behavior Science (IF = 3.8), Journal of the Experimental Analysis of Behavior (JEAB; IF = 1.9), Behavior Analysis in Practice (IF = 1.7), Education and Treatment of Children (IF = 1.4), The Analysis of Verbal Behavior (IF = 0.8), and The Psychological Record (IF = 0.8).*

### Database searches

Searches within each of the specified journals were initially undertaken in March 2023 using APA PsycINFO via the ProQuest electronic database. The search was updated in December 2024 to capture the full publication record for 2023. We selected this date to ensure complete coverage for a full year for all journals (for the purpose of detecting trends over time). The following search string was used to identify eligible studies: publication(“Education and Treatment of Children”) AND tiab(“meta-analy*” OR “systematic review”). We adapted the search string for each journal by replacing the publication specifier with the respective journal name. Once duplicates had been removed, the list of studies was recorded on an Excel spreadsheet. To determine eligibility, two graduate-level reviewers independently reviewed the titles and abstracts in accordance with the study inclusion criteria and classified papers as include, exclude, or uncertain. The reviewers consulted the full text of the paper for all studies classified as either include or uncertain. Any disagreements were resolved via discussion with a third member of the research team.

### Quality assessment

We assessed all included studies using the AMSTAR 2 and R-AMSTAR instruments. The AMSTAR 2 consists of 16 items that are evaluated with “yes” or “no” (1, 3, 5, 6, 10, 13, 14, 16); with “yes”, “partial yes”, or “no” (2, 4, 7, 8, and 9); or with “yes”, “no”, or “no meta-analysis conducted” (11, 12, and 15). A summary of each item is presented in the results; however, see the appendix for a full description of each item. Items 2, 4, 7, 9, 11, 13, 15 are deemed to be “critical” as they have a substantial impact on the confidence regarding the validity of a review and its conclusions [18]. Scoring an item as “yes” indicated that a particular review had met the item criterion.

The developers of the AMSTAR 2 explicitly recommend that individual item ratings are not combined to create an overall score [18]. We therefore employed the R-AMSTAR instrument developed by Kung et al. [19] as a means of quantifying overall methodological quality. The R-AMSTAR is based on the original AMSTAR 11-item tool; however, each of the items has been broken down into sub-criteria which a review either meets or does not meet, with some items marked “not applicable” when a meta-analysis was not conducted. We anticipated a high proportion of reviews containing SCEDs, therefore we used a version of the R-AMSTAR that is better suited to evaluating reviews and meta-analyses that include SCEDs [21]. For every review, we coded whether the study met each of the 44 criteria in the instrument (four criteria from each of the 11 items). If a study explicitly met an individual criterion, we gave it a score of 1, otherwise we assigned it 0 (e.g., for ‘no’, ‘not reported’ or ‘not applicable). We then calculated the total score by summing the scores of all criteria that were met. For reviews that included a meta-analysis, the maximum achievable score was 44, for reviews that did not include a meta-analysis the maximum achievable score was 41. For the purposes of descriptive reporting, scores were converted to a percentage of the maximum applicable score for each study, allowing comparison of overall adherence across reviews regardless of whether a meta-analysis was conducted. In the statistical analysis, this variation in the number of applicable items was accommodated within the model. Each item of the R-AMSTAR was modeled as an individual Bernoulli trial, with the differing denominators handled through the model structure rather than through score standardisation. The item-level criteria for the modified R-AMSTAR are shown in the results section.

Two researchers independently scored all the included studies with both instruments. When there was a disagreement with respect to scoring, the item was independently reviewed and determined by a third member of the research team. For both primary measures, inter-rater reliability was assessed using overall percentage agreement and Gwet’s AC1. For items where ‘Not Applicable’ was a valid response (Item 9), or where data were missing, these were treated as a distinct third category to ensure that disagreements regarding item applicability were captured in the reliability estimate. For the R-AMSTAR, overall inter-rater agreement was calculated as 81.7% and mean Gwet’s AC1 was 0.73. For the AMSTAR 2 ratings, inter-rater agreement was 88.8%, and Gwet’s AC1 was 0.86. The items that generated the most common disagreement between raters were whether the adequate level of study detail was included (Item 8) whether satisfactory risk of bias assessment was undertaken in the primary studies (Item 9)

### Study characteristics

The following data were extracted for each paper where it was available: (a) author and year of publication); (b) country of institutional affiliation of all authors (c) intervention characteristics (if appropriate) (d) primary outcome; (e) the number of included studies; (f) type of experimental designs included (if appropriate); (g) participants characteristics; and (h) presence of a PRISMA statement. In addition to these items, all of which were specified in our preregistration, we also recorded whether the review, (i) covered an intervention or procedure that was applied in nature (i.e., had social significance for the recipients). Data from each paper were independently extracted by two members of the research team. Where we encountered a discrepancy between the two reviewers, a third person reviewed the item and came to a decision. We calculated total Interobserver Agreement (IOA) for items (c) through (i), by dividing number of agreements by the total number of items scored. This resulted in an overall IOA score of 82.8%. Consistent with the approach adopted in systematic reviews that report percentage agreement, this exceeded the commonly used threshold of 80% [25].

### Outcome variables and planned analysis

- To determine the methodological quality of the included systematic reviews we used the AMSTAR 2 [18] to score each of the included reviews. For each of the 16 items, we provided a narrative synthesis.
- Using the AMSTAR 2, we rated the included reviews as “High”, “Moderate”, “Low” and “Critically Low” in terms of their methodological quality. We also present the number of “Critical Items” (from those eligible items) scored as “No”. Eligibility for items relevant to a meta-analysis was determined by whether the review included a meta-analysis (yes/no).
- We also calculated a score for each study using an adapted version of the Revised AMSTAR (R-AMSTAR) [19]. The adaptations include the scoring amendments detailed by Jamshidi et al. [21].

### Statistical analysis

In order to examine change in scores over time we used a hierarchical logistic regression modelling approach. Regression analysis has been used in previous studies to explore changes in R-AMSTAR adherence across time [21]. Here, as specified in our preregistered analysis plan we employed a Bayesian regression framework. In a Bayesian approach, prior information about the parameters in the model (e.g., priors) are specified before the observed data are incorporated. Combining these prior parameter distributions with the observed data yields a posterior distribution for each parameter. Statistical inferences are then drawn by examining the central tendency and spread of the posterior distributions. Specifying priors allows reasonable assumptions to be incorporated into the model, constraining estimates to plausible ranges [26]. We used mildly informative, regularising priors, which encode skepticism toward extreme values and help prevent over-fitting to outliers in the data.

Each R-AMSTAR item was coded dichotomously (1 = criterion met; 0 = not met). To accommodate the variation in numbers of eligible items (e.g., meta-analysis-specific items that were not applicable), we modeled each item as one Bernoulli trial and allowed random intercepts for both study and journal which accounted for the varying number of applicable items across studies. The model specification was as follows: let *i* index items, *j* studies, and *k* journals where *j[i]* and *k[i]* denote the study and journal associated with item response *i*. With *y*_*i*_ ∈{0,1} and mean-centered publication year *x*_*i*_,


$$\begin{array}{c} {\text{y}}_{\text{i}}\sim \text{Bernoulli}\left({\text{p}}_{\text{i}}\right) \end{array}$$

$$\begin{array}{c} ogit\left(p_{i}\right)=\beta_{\text{0}}+\beta_{\text{1}}\;x_{i}+u_{\;j[i]}+v_{k[i]} \end{array}$$

$$\begin{array}{c} u_{j}\sim N\left(\text{0},\;\sigma_{\text{study}}\right) \end{array}$$

$$\begin{array}{c} v_{k}\sim N\left(\text{0},\;\sigma_{\text{journal}}\right) \end{array}$$

$$\begin{array}{c} \beta_{\text{0}}\sim N(\text{0},\;\text{3}) \end{array}$$

$$\begin{array}{c} \beta_{\text{1}}\sim N(\text{0},\;\text{0}.\text{2}\text{5}) \end{array}$$

$$\begin{array}{c} \sigma_{\text{study}},\;\sigma_{\text{journal}}\sim \text{Exponential} \end{array}$$ (1)

Thus, the model includes (1 | study) and (1 | journal) random intercepts and a single population-level slope for year (i.e., average yearly improvement across studies). We used weakly informative priors: Normal(0, 3) for the intercept (allowing a wide range on the probability scale), Normal(0, 0.25) for the year effect, and Exponential (1) for the standard deviations. Before fitting to data, we examined whether our priors implied reasonable outcomes by conducting prior-only simulations (i.e., simulating from the prior predictive distribution). We also performed generative simulations to verify that the model recovered (known) parameters. Specifically, we created synthetic data sets with the same hierarchical structure (Bernoulli outcomes with random intercepts for study and journal, and a fixed effect of centered year), varying the true year slope across multiple scenarios (e.g., none/moderate/strong). We chose this analytic approach for two reasons: First, hierarchical models employ partial pooling, whereby estimates for individual journals are informed both by journal-specific data and by the overall distribution of effects across journals. This is particularly beneficial when the number of studies nested within individual journals is small, as it prevents the model from over-relying on sparse local data while still allowing journal-level variation to be estimated. Second, the Bayesian framework affords greater flexibility in handling convergence with small sample sizes, a consideration that was particularly pertinent given that, at the point of preregistration, the sample sizes nested within individual journals were not yet known.

The models were fitted using the Stan computational framework (http://mc-stan.org) via the brms package [27] accessed through the statistical computing software R [28]. Model estimation was performed using Markov Chain Monte Carlo via the No-U-Turn Sampler [29]. The models were run with four parallel chains with 2000 iterations including a 1000 iteration warm up, resulting in 4000 post-warmup samples. The quality of the models was assessed by inspecting the chains to ensure mixing, convergence and stationarity. We verified that the number of effective samples was above 200 and that Rhat values were approximately 1. The data and code used to perform all the statistical analysis is provided at the following Open Science Framework repository: https://doi.org/10.17605/OSF.IO/QRGEW.

The figures were produced using the dplyr [30] and ggplot2 [31] packages. We also used the marginaleffects package [32] to generate the marginal effect estimates. To represent the central tendency and spread of the posterior distributions we report the posterior mean estimates and their corresponding 95% credible intervals (CI).

### Deviations from preregistration

We deviated from the planned analyses in two ways. Following the recommendations of Willroth and Atherton [33], we describe each deviation, its rationale, and likely consequences for the study’s conclusions.

The first deviation was the omission of the planned AMSTAR 2 regression model. We had initially intended to use both AMSTAR 2 and R-AMSTAR scores as outcome variables in separate regression analyses. Upon closer consideration of AMSTAR 2, we determined that aggregating items into a total score was inappropriate, as the instrument’s developers explicitly recommend against quantitative summation for inferential purposes [18]. This decision was methodological rather than data-contingent. The deviation is unlikely to have biased our conclusions: R-AMSTAR was explicitly designed for quantitative interpretation and has been used for this purpose in prior work [21], while the AMSTAR 2 findings are reported in full descriptively and converge with the R-AMSTAR results.

The second deviation was the omission of the planned subgroup comparison between reviews involving exclusively neurodiverse participants (autistic individuals or those with intellectual or developmental disabilities) and those involving exclusively neurotypical participants. In the final dataset, very few reviews met these criteria, rendering the planned comparison statistically uninformative. Although this deviation narrows the scope of inference, it is unlikely to have materially affected our primary conclusions regarding methodological quality and temporal trends.

## Results

**Fig 1:**
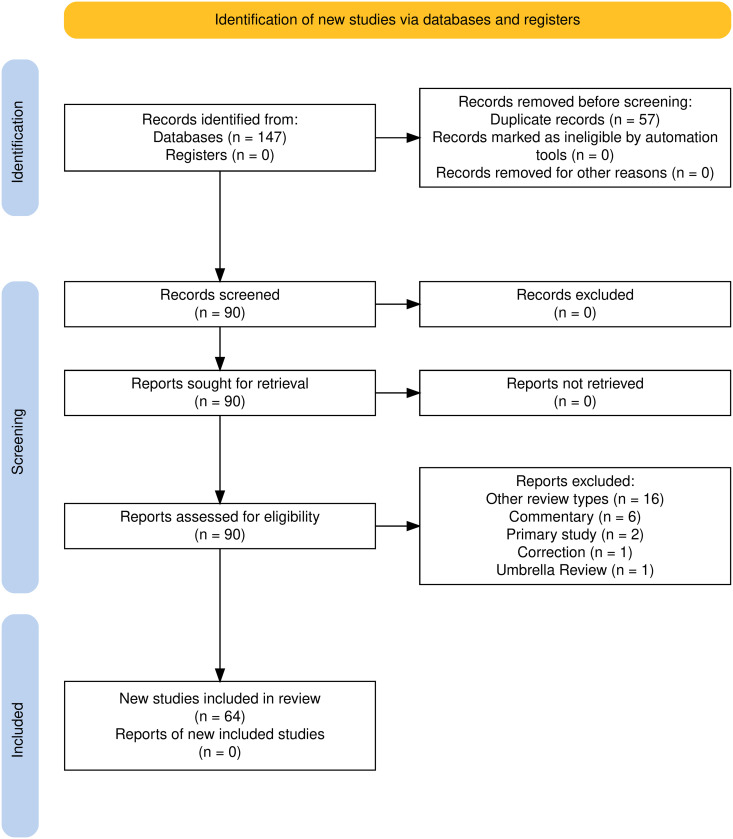
Preferred Reporting Items for Systematic Reviews and Meta-analyses (PRISMA) flow diagram of decision process for included studies.

The initial electronic database search produced 145 records. This list was screened for duplication resulting in 88 unique records. The initial screening of the studies resulted in 87 agreements (98.9.% agreement, κ = 0.97). The single disagreement was resolved via discussion and consultation with a third member of the research team. We used the same process for the updated search which was designed to capture all studies published in 2023. We identified two additional studies in the updated search, resulting in 90 papers that were screened for eligibility in total. Agreement for the updated searches was 100%. Following screening, 64 papers were selected for inclusion in the review (the process is summarised in Fig 1 using the PRISMA flowchart). All studies identified as meeting the eligibility criteria were published in English. The full list of the studies included in (and excluded from) the review are available in the supplementary materials.

**Table 1:** Methodological quality of systematic review according to R-AMSTAR modified criteria.

R-AMSTAR items	Criteria	Total studies met (%)	Mean (Median)
1. Was an “a priori” design provided? The research questions/objectives and inclusion criteria should be established a priori as evidenced by a published protocol or an explicit statement in the review.	(A) Study protocol or ‘a priori’ design is published and/or registered in advance (B) Description of inclusion criteria (C) A clearly focused (PICO-based) question	A) 2 (3.1%) (B) 47 (73.4%) (C) 11 (17.2%)	Mean: 0.31 Median: 0.33
2. Was there duplicate study selection and data extraction?	(A) At least two persons independently selected studies, explicitly stated (B) Statement of consensus procedure for disagreements in selecting studies (C) Disagreements among extractors resolved properly as stated or implied in selecting studies (D) At least two persons independently extracted the data, explicitly stated (E) Statement of consensus procedure for disagreements (F) Disagreements among extractors resolved properly as stated or implied	(A) 44 (68.8%) (B) 23 (35.9%) (C) 37 (57.8%) (D) 50 (78.1%) (E) 37 (57.8%) (F) 42 (65.6%)	Mean: 0.61 Median: 0.67
3. Was a comprehensive literature search performed?	(A) At least two electronic sources are searched (B) Publishing time range of the included studies are mentioned (C) Used databases are mentioned (D) Key words and/or MeSH terms are stated and where feasible the search strategy outline is provided (E) Searches should be supplemented by consulting current contents, reviews, textbooks, the reference lists of eligible studies, citation indices, contacting experts (F) Journals are hand-searched or manually searched	(A) 55 (85.9%) (B) 47 (73.4%) (C) 64 (100.0%) (D) 61 (95.3%) (E) 52 (81.2%) (F) 18 (28.1%)	Mean: 0.77 Median: 0.83
4.Was the status of publication (i.e., grey literature) used as an inclusion criterion?	(A) The authors stated that they searched for reports regardless of their publication type (B) The authors stated whether or not they excluded any reports based on their publication status, language etc. (C) “Non-English papers were translated” or readers sufficiently trained in foreign language (D) No language restriction or recognition of non-English articles	(A) 17 (26.6%) (B) 50 (78.1%) (C) 1 (1.6%) (D) 8 (12.5%)	Mean: 0.30 Median: 0.25
5.Was a list of studies (included and excluded) provided?	(A) Table/list/figure of included studies, a reference list does not suffice (B) Table/list/figure of excluded studies either in the article or in a supplemental source (C) Satisfactory/sufficient statement of the reason for exclusion of the seriously considered studies (D) Reader is able to retrace the included and the excluded studies anywhere in the article bibliography, reference or supplemental source	(A) 62 (96.9%) (B) 1 (1.6%) (C) 30 (46.9%) (D) 2 (3.1%)	Mean: 0.37 Median: 0.25
6.Were the characteristics of the included studies provided?	(A) In an aggregated form such as a table, data from the original studies are provided on the participants, interventions/exposure, outcomes,... the ranges of characteristics in all the studies analyzed, e.g., age, race, sex, relevant socioeconomic data, disease status, duration, severity, or other diseases should be reported. (B) Ranges are provided of the relevant characteristics in the analysed studies (C) The information provided appears to be complete and accurate	A) 53 (82.8%) (B) 27 (42.2%) (C) 60 (93.8%)	Mean: 0.73 Median: 0.67
7.Was the scientific quality of the included studies assessed and documented?	(A) ‘A priori’ methods of assessment are provided (B) The scientific quality of the included studies appears to be meaningful (C) Discussion/recognition/awareness of level of evidence is present (D) Quality of evidence is rated/ranked based on characterized instruments over all included studies (E) Quality of evidence is rated/ranked based on characterized instruments for each individual study	(A) 2 (3.1%) (B) 8 (12.5%) (C) 26 (40.6%) (D) 22 (34.4%) (E) 22 (34.4%)	Mean: 0.25 Median: 0
8.Was the scientific quality of the included studies used appropriately in formulating conclusions?	(A) The scientific quality is considered in the analysis and the conclusions of the review (B) The scientific quality is explicitly stated in formulating recommendations (C) Conclusions integrated/derived towards practice guidelines and recommendations	(A) 22 (34.4%) (B) 20 (31.2%) (C) 43 (67.2%)	Mean: 0.44 Median: 0.33
9.Were the methods appropriately used to combine the findings of studies?	(A) Statement of criteria that were used to decide that the studies analysed were similar enough to be pooled (B) For the pooled results, a test is done to ensure the studies were combinable, to assess their homogeneity (C) A recognition of heterogeneity or lack thereof is present (D) If heterogeneity exists a ‘random effects model’ is used and/or the rationale (i.e., clinical appropriateness) of combining should be taken into consideration (i.e., is it sensible to combine?), or stated explicitly	^a^(A) 10 (15.6%) (B) 5 (31.2%) (C) 8 (50.0%) (D) 6 (37.5%)	Mean: 0.15 Median: 0
10.Was the likelihood of publication bias assessed?	(A) Recognition of publication bias or file-drawer effect (B) An assessment of publication bias should include graphical aids (e.g., funnel plot, other available tests) (C) Statistical tests (e.g., Egger regression test)	(A) 24 (37.5%) (B) 1 (1.6%) (C) 5 (7.8%)	Mean: 0.16 Median: 0
11.Were conflicts of interest disclosed for all of the review authors and was the funding source of the review and of each study within the review reported?	(A) Statement of sources of support (B) No conflict of interest is mentioned (C) An awareness/statement of support or conflict of interest in the primary included studies	(A) 16 (25.0%) (B) 30 (46.9%) (C) 0 (0.0%)	Mean: 0.24 Median: 0.33

^a^Item 9 criteria B-D apply only to reviews that pooled results (n=16); criterion A was assessed across all reviews (n=64). Item scores were used using each reviews applicable criteria.

### Study characteristics

Of the 64 systematic reviews we identified, almost half were published in *Education and Treatment in Children* (43.8%). Eleven were published in the *Journal of Applied Behavior Analysis* (17.2%), and nine in both *Behavior Analysis in Practice* (14.1%) and *Perspectives on Behavior Science* (14.1%). The remaining 11% of reviews were published in *The Psychological Record* (*k* = 3), *The Analysis of Verbal Behavior* (*k* = 2) and *Journal of the Experimental Analysis of Behavior* (*k* = 2). The included studies were published between 1988 and 2023, with a median publication year of 2020. There was a notable increase in publications from 2018 onward, with 67.2% (*k* = 43) of the studies published in the period between 2018 and 2023. Authors from institutions in the USA were represented in the majority of studies (86.6%, *k* = 58). Canadian institutions were represented in four studies, while institutions from Wales, Australia, South Korea, and New Zealand each contributed two studies. The remaining studies included authors from institutions in Northern Ireland, Republic of Ireland, India, Belgium, and Colombia.

A wide variety of topics were covered (see supplementary materials for details of individual studies) including intervention (79.7%; *k* = 51) and non-intervention-focused (20.3%; *k* = 13) topics. An example of a non-intervention review, Odum et al. [34], reviewed delay discounting literature, whereas an intervention review example included Hawken et al., [35], who reviewed the evidence for the *Check-in/Check-out* intervention in schools. Of the eligible reviews, (64.1%; *k* = 41), involved autistic participants, or individuals with intellectual or developmental disability (this included individuals described as having a learning disability, Down Syndrome, or Pervasive Developmental Disorder); however, only 11 reviews (17.2%) comprised samples drawn exclusively from these populations. Some of the reviews did not provide information about the study participant demographics. For example, a review on publication practices for which participant information was not applicable [36], and three papers in which participant information was not explicitly reported [34,37,38].

The number of included studies within the reviews ranged from 6 to 200 (median = 23) and 25% of the studies involved a meta-analysis (*k* = 16). Of the research designs included in the primary studies 78.1% (*k* = 50) reported using single case experimental designs, and 46.9% (*k* = 30) included studies with some type of group-based design or analysis (e.g., Randomised controlled trial (RCT); quasi-experimental group design, within-group, correlational). Six studies (9.4%) either did not report primary study designs, reported information was unclear, or this information was not applicable given the topic of the review. PRISMA guidelines were used by 39.1% (*k* = 25) of the included reviews. More than three quarters of the reviews did not include any information about a funding source for the review (76.6%). Of those that mentioned a funder, 17.2% reported funding for the review and 6.2% reported no funding.

### Quality assessment: AMSTAR 2

**Fig 2:**
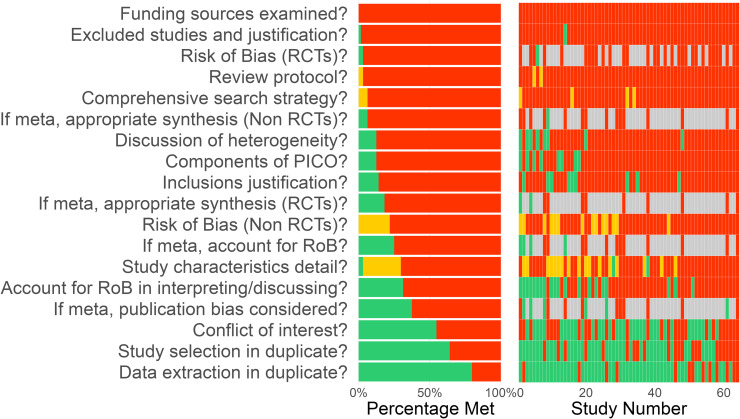
AMSTAR 2 ratings for all included reviews. Green indicates “Yes” (criterion fully met), orange indicates “Partial” (criterion partially met), red indicates “No” (criterion not met), and grey indicates “Not Applicable” (criterion not relevant for that review; for example, if no meta-analysis was performed). The items on the left are presented in order of adherence (or partial adherence) to the AMSTAR 2 from lowest (at the top) to highest. Note. PICO = participant, intervention, comparison, outcome; RoB = risk of bias; RCT = randomised controlled trial; meta = meta-analysis.

The AMSTAR 2 results are shown in Fig 2 and are included in full in the supplementary materials. The bar chart (left) shows the percentage of eligible systematic reviews that met each AMSTAR-2 assessment criterion, calculated only from reviews where the criterion was applicable. Items are ordered by agreement level, with the lowest agreement items at the top of the plot. The heatmap (right) displays individual review responses for each criterion, with studies ordered by the cumulative number of ‘Fully met’ and ‘Partially met’ responses, from the highest to lowest (from left to right). This visualisation reveals both overall patterns of methodological quality across the different items of the AMSTAR 2 for the sample of studies as a whole, but also the study-specific variations in adherence.

In only three of the AMSTAR 2 items (item 5, 6, and 16) did more than 50% of the reviews fully meet the criterion. The most commonly adhered items were Item 6 (79.7%; *Authors performed data extraction in duplicate)* followed by Item 5 (64.1%; *Authors performed study selection in duplicate)* and then Item 16 (54.7%; *Authors reported any potential sources of conflict of interest, including any funding they received for conducting the review)* The items that performed least well were as follows: 1) the presence of a review protocol (Item 2; partially met in 3.1% of reviews); 2) listing and justification for excluded studies (Item 7, fully met criterion in 1.6% of studies); 3) Assessing Risk of Bias for studies involving RCTs (Item 12, fully met criterion in 3.1% of studies); 4) Performing a fully comprehensive search strategy (Item 4, partially met in 6.2%); and 5) Reporting on sources of funding in the primary studies (Item 10, not met in any study).

The overall methodological quality of the included studies was rated according to criteria specified in Shea et al., [18]. For a determination of high confidence in the results a review should have either no or one non-critical weakness. For a rating of moderate confidence, a review should have no critical flaws. For a high confidence rating, a review should have no more than one non-critical weakness. Finally, for a methodological quality rating of ‘critically low’, a review contains two or more critical flaws. All studies in our review received a rating of “critically low” indicating that each contained more than one critical flaw (with or without non-critical weaknesses).

The most common critical flaws across the studies were Item 2 (0%; *Protocol registered before commencement of the review*), Item 4 (0%; *Adequacy of the literature search)*, Item 11 (0%; *Appropriateness of meta-analytical methods;* overall score from both RCTs and Non-RCTs) and Item 9 (0%; *Risk of bias from individual studies being included in the review*; overall score from both RCTs and non-RCTs). No reviews scored ‘yes’ on any of these items. The critical items that were adhered to most often were Item 15 (40%; *Assessment of presence and likely impact of publication bias),* followed by Item 13 (31.2%; *Consideration of risk of bias when interpreting the results of the review*), and finally Item 7 (1.6%; *Justification for excluding individual studies)*.

### Quality assessment: R-AMSTAR

**Fig 3:**
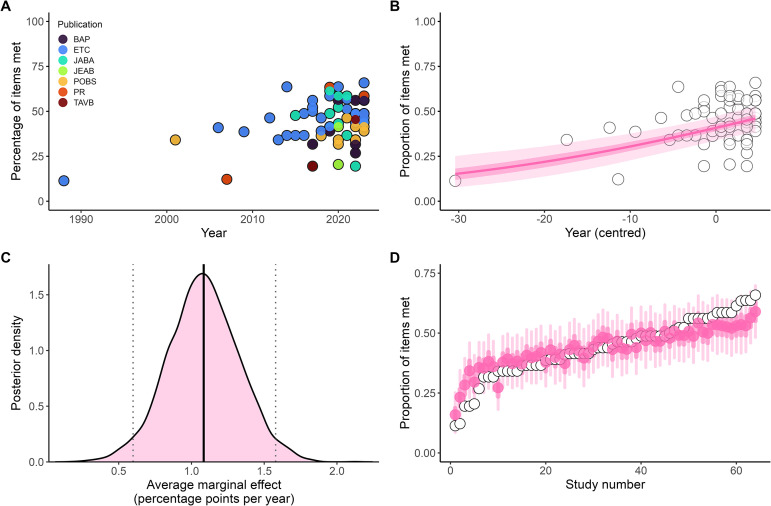
R-AMSTAR compliance across systematic reviews, and Bayesian hierarchical model estimates of change over time. **(A)** R-AMSTAR compliance rates by publication and year. Each point represents an individual systematic review, with colours indicating the publication source; the y-axis shows the percentage of R-AMSTAR criteria met by each review. **(B)** Predicted probability of R-AMSTAR item adherence over time. The solid pink line shows the population-average predicted probability of meeting individual R-AMSTAR criteria as a function of mean-centred publication year, derived from a Bayesian hierarchical logistic regression model with random intercepts for studies and journals; the darker and lighter pink ribbons show the 50% and 95% credible intervals, respectively, and the open circles represent observed (raw) study-level adherence proportions. **(C)** Posterior distribution of the average marginal effect (AME) of a one-year increase on R-AMSTAR adherence, expressed in percentage points per year. The density represents the posterior distribution of the yearly change in probability of meeting individual R-AMSTAR criteria; the solid black line marks the mean AME (1.08 percentage points per year) and the dotted lines mark the 95% credible interval (2.5th and 97.5th percentiles of the posterior). **(D)** Model fit assessment: predicted versus observed R-AMSTAR adherence. Studies are ordered from lowest (left) to highest (right) observed adherence proportion; open circles represent observed study-level proportions, filled pink circles show model-predicted values, and pink vertical lines indicate 95% credible intervals around predictions. *Note: BAP = Behavior Analysis in Practice; ETC = Education and Treatment of Children; JABA = Journal of Applied Behavior Analysis; JEAB = Journal of the Experimental Analysis of Behavior; POBS = Perspectives on Behavior Science; PR = Psychological Record; TAVB = The Analysis of Verbal Behavior.*

We scored each review against the 44 criteria specified in the R-AMSTAR (41 for studies without a meta-analysis). Table 1 details the percentage of reviews that met each criterion on an item-by-item basis (see supplementary materials for study-level scoring). For each review we then calculated the total number of R-AMSTAR criteria that had been met and divided that by the total number of eligible items. This gave us a percentage score for each study. This study-level raw percentage data can be seen in Fig 3A.

We then used the R-AMSTAR scores to answer our second research question: Has adherence to methodological and reporting standards in systematic review improved across time? The multilevel logistic regression revealed a positive temporal trend in R-AMSTAR adherence (β = 0.05, 95% CI: [0.02, 0.07]), indicating that the log-odds of meeting each criterion increased by 0.05 per year. This corresponds to an odds ratio of 1.05 (95% CI: [1.02, 1.07]), or approximately a 5% increase in the odds of compliance per year. To express this effect in more interpretable terms, the average marginal effect was 1.08 percentage points per year (95% CI: [0.59, 1.58]), meaning that, across studies, the probability of meeting R-AMSTAR criteria increased by approximately 1.08 percentage points annually. Fig 3B displays the model-predicted probability of meeting R-AMSTAR criteria as a function of publication year.

Fig 3C shows the full posterior distribution of the average marginal effect (AME). The entire posterior distribution lies above zero, which provides robust evidence for improvement in systematic review quality over the study period. We observed moderate variability between journals in their average quality levels (SD = 0.27 on the log odds scale) for journal random intercepts; however, the limited number of studies within each journal (2–28, with four journals having fewer than 5 studies) means that this estimate should be treated with caution. A greater number of studies per journal would have allowed us to better detect any systematic differences. The variation between individual studies (SD = 0.31 on the log odds scale) was comparable to or slightly larger than the variation between journals, suggesting that study-level factors may be as important as journal-level factors in determining adherence.

Model fit was assessed by comparing predicted and observed study-level R-AMSTAR adherence rates (Fig 3D). Studies are ordered by raw percentage of items met from lowest to highest. The hierarchical structure induced shrinkage toward the population mean for extreme values. The model’s tendency to predict values slightly closer to the population average than observed extreme values demonstrates the regularising effect of the hierarchical structure, which borrows information across studies and publications to improve overall prediction quality. The 95% credible intervals appropriately captured the observed variation, indicating well-calibrated model predictions.

### Exploratory analysis

In the design of our preregistered model, we attempted to build in robustness to the influence of outliers. Specifically, the hierarchical structure of the model provides inherent protection against the undue influence of individual reviews or journals; partial pooling across studies and journals shrinks estimates toward the overall mean which attenuates the leverage of outliers. Nevertheless, to further interrogate the robustness of the observed temporal trend, we conducted sensitivity analyses examining: (1) whether improvement was driven by the sparse early reviews and (2) whether a non-linear temporal pattern better fit the data.

First, we re-fitted the hierarchical logistic regression model excluding reviews published before 2010 (k = 5 studies removed, leaving k = 59 post-2010 reviews). This tested whether the observed improvement was primarily driven by particularly low adherence in early reviews rather than reflecting consistent progress across the entire study period. Results: The positive temporal trend persisted when restricting to post-2010 data (average marginal effect = 0.7 (95% CI: [−0.32, 1.67]) percentage points per year. So, while the magnitude of the effect was somewhat attenuated, the direction of the effect remained consistent with the full-sample analysis, indicating that improvement was not solely attributable to early outliers.

Second, we fitted a Bayesian hierarchical Generalized Additive Model (GAM) with a smooth term for year (thin plate regression splines, k = 5 basis functions) to allow the data to reveal non-linear patterns. This addressed whether improvement was accelerating, decelerating, or otherwise non-linear over time. The average marginal effect from the GAM was 0.93 (95% CI: [0.05, 1.64]) percentage points increase per year, which was broadly consistent with the findings of the original logistic hierarchical model estimate. Model comparison using leave-one-out cross-validation (LOO-IC) revealed no meaningful difference between the linear and GAM specifications (ΔLOO = −0.2, SE = 0.5), indicating that the linear model adequately captured the temporal trend without requiring additional flexibility. These sensitivity analyses collectively demonstrate that: (1) the temporal improvement in R-AMSTAR adherence is present across the full range of years included in the study; (2) the relationship between year and adherence is genuinely linear rather than constrained by our modelling choices.

We conducted a further sensitivity analysis excluding 12 reviews that focused on non-intervention or basic research topics, retaining only those studies that evaluated interventions or procedures related to socially significant outcomes (k = 52). We maintained the same random effects structure and priors as in the primary analysis. The results were consistent with the primary analysis: the average marginal effect of publication year on R-AMSTAR adherence was 1.15 percentage points per year (95% CI [0.69, 1.62]). This suggests that the inclusion of non-intervention reviews did not substantially influence our conclusions regarding temporal trends in methodological quality.

Finally, we examined whether methodological quality differed between reviews that included a meta-analysis and those that did not. Specifically, meta-analytic status was included as a binary fixed effect in the multilevel Bernoulli model alongside publication year, retaining the same random effects structure and priors as the primary analysis. A weakly informative normal prior (mean = 0, SD = 0.5) was specified for the meta-analytic status coefficient, which served to regularise estimates toward zero while permitting credible effects of plausible magnitude. The predicted probability of meeting any given criterion was 38.9% (95% CI: 31.2–45.5%) for non-meta-analytic reviews and 46.8% (95% CI: 38.1–55.1%) for meta-analytic reviews, a difference of 8.0 percentage points (95% CI: 1.6–14.2%). This suggests that reviews incorporating a meta-analysis tend to demonstrate modestly higher methodological quality than those that do not.

## Discussion

The present study investigated, for the first time, the overall methodological quality of systematic reviews and meta-analyses published in key behaviour analysis journals. Studies were rated according to widely adopted criteria specified in AMSTAR 2 and the R-AMSTAR instruments. We found that all eligible studies were rated as being of ‘critically low’ quality because they contained more than one ‘critical flaw’. According to AMSTAR 2 guidance, a review that receives this rating should not be relied upon to provide an accurate and comprehensive summary of the available evidence. The R-AMSTAR assessment, which we used to generate a percentage compliance score, revealed that studies adhered to, on average, 43.8% of items on the R-AMSTAR (range 11% − 66%). We then investigated the extent to which adherence improved across time and found evidence of gradual but robust improvement. These findings indicate that, while methodological standards remain variable, there is credible evidence of a gradual shift toward more rigorous practices.

All of the studies that we reviewed (*k* = 64) were deemed ‘critically low’ based on the AMSTAR 2 classification. A critically low rating indicates that the way in which the review has been undertaken and/or reported is not sufficiently robust to infer that the findings are accurate or comprehensive. A review receiving this rating may have failed to register a protocol prior to commencement, conduct comprehensive literature searches, justify study exclusions, assess risk of bias in included studies, employ appropriate meta-analytical methods, assess publication bias, or consider bias when interpreting results, all of which are deemed by AMSTAR 2 to be fundamental components of reliable evidence synthesis. Notably, a number of ‘critical’ items were not met by any of the eligible reviews. For example, no study fully satisfied the criteria for study protocol registration (Item 2). Only two studies partially met the criteria, with the remaining studies failing to provide any evidence of protocol registration. Protocol registration in publicly accessible repositories increases transparency and prompts researchers to clearly define research questions and intended analyses in advance of undertaking the review. Taking the steps to register a study protocol in advance protects against retrospective interpretation of findings in ways that favour positive results or post-hoc theorizing, thereby enhancing the credibility of the research [39]. A recent study by Ge et al. [40] examined the impact of protocol registration and found those that reviews reporting a preregistered protocol tended to be of higher quality.

Protocol registration has long been regarded as a gold standard practice. A study of reviews of intervention studies published between 2020 and 2021 found that 38% provided information about a registered protocol [41]. Indeed, the original PRISMA statement [17], published in 2009, recommended reviews report registration information, and the Prospective Register of Systematic Reviews (PROSPERO), which provides researchers with an accessible platform for protocol registration, was launched in 2011. Despite the availability of these resources and established norms in other fields for over a decade, adoption within behaviour analysis seem to be rare (i.e., 3% of the present sample). This mirrors the findings of Jamshidi et al. [21] who found no evidence of protocol registration in a review of 178 meta-analyses in the single-case experimental design literature. The absence of protocol registration suggests that targeted efforts are needed to promote awareness and implementation within the behaviour analysis community.

Encouragingly, developments in the field suggest growing recognition of these issues. For example, Tincani et al. [42] recently recommended the adoption of preregistration in behaviour analysis research.They acknowledged that although such practices remain underused within the field, the benefits (e.g., enhanced methodological rigour and transparency) outweigh the associated costs. Tincani and colleagues call for systemic change, urging journal editors and field leaders to establish policies and contingencies that promote preregistration and other open science practices. Our findings underscore the need for coordinated efforts to bridge the gap between established methodological standards and current practices within behaviour analysis. The convergence of empirical evidence documenting low adoption rates and emerging calls for change may represent a critical juncture for advancing open science practices within the field [43].

Adequate risk of bias assessment was absent from all but one study in the present sample. Risk of bias assessment involves the systematic evaluation of methodological limitations and biases of included studies, including selection bias, performance bias, detection bias, and reporting bias. Risk of bias assessment has long been considered fundamental to systematic review methodology as it directly impacts the validity and reliability of synthesised conclusions. Without such information, readers cannot gauge whether the review’s findings might be undermined by methodological weaknesses in the underlying evidence. The widespread absence of sufficiently comprehensive risk of bias assessment is therefore particularly concerning. While tools like ROBIS (Risk of Bias in Systematic Reviews) [44] are widely used elsewhere, they are clearly rarely used in this body of literature.

The absence of risk of bias assessments was surprising given the proportion of studies (39%) that cited using the PRISMA reporting guidelines [4,17]. Risk of bias assessment and protocol registration both feature in the PRISMA checklist. This discrepancy suggests there is a gap between stated adherence to established guidelines and their actual implementation. This pattern is consistent with what has been documented in other fields. For instance, Ivaldi et al. [45] found that the average adherence to items in the PRISMA 2020 statement in published systematic reviews was around 42%. One possibility for the low adherence to risk of bias evaluation in the present study is that risk of bias instruments are less well-developed for single-case experimental designs, which are commonly used in behaviour-analytic research. This argument, however, fails to explain the absence from studies that included group-based experimental designs, for which established instruments are available. Furthermore, specialized tools such as the Risk of Bias in N-of-1 Trials Scale (RoBiNT) [46] and Single Case Design Risk of Bias Tool [47] are available for single-case research. Our findings raise important questions about the role of journal editors and peer reviewers in ensuring adherence to stated reporting guidelines, particularly when authors explicitly claim to follow established frameworks like PRISMA.

Among studies that did report on risk of bias, various tools were utilised. This included the What Works Clearinghouse (WWC) standards, which provides quality standards for both group and single-case designs [48], the CEC’s Standards [49], and the Evaluative Method for Determining EBP in Autism [50]. Where an instrument was used, it was not always sufficient to address the AMSTAR 2 requirements. For nonrandomised studies to receive a score of “Yes” on Item 9 (*Did the review authors use a satisfactory technique for assessing the risk of bias (RoB) in individual studies that were included in the review?*), reviews would be required to assess risk of confounding, selection bias, methods used to ascertain exposures, and risk of the outcomes being selectively reported. These domains derive from ROBINS-I [51], which is widely regarded as the most comprehensive and rigorous quality appraisal tool, and the instruments used in the reviews varied in how fully they addressed them. AMSTAR 2 places particular emphasis on comparing reported analyses with preregistered protocols when appraising risk of selective reporting, for example, and tools such as the Evaluative Method for Determining EBP in Autism [50] address this domain only partially. Reviews relying on this instrument therefore fell short of meeting Item 9, despite having conducted a formal risk-of-bias appraisal. The differences in quality appraisal tools reflect differing views on what makes a study methodologically robust.

Our finding that key methodological features are frequently missing in this literature has important implications for the field of behaviour analysis. As systematic reviews constitute a fundamental source of evidence for informing intervention selection and implementation, neglect of best practices in evidence synthesis may lead to several unintended consequences. Decision-makers who rely on reviews with substantial methodological gaps may inadvertently select less effective interventions while overlooking more promising alternatives. Clearly, this has implications for those receiving services and the judicious allocation of resources. Furthermore, published but methodologically weak reviews may acquire disproportionate influence: they may be perceived as authoritative due to their systematic review designation, obscuring their methodological weaknesses. Finally, these issues also contribute to research inefficiency. As funding bodies and commissioning organisations increasingly mandate adherence to contemporary methodological standards, reviews that fail to meet these criteria become less useful for decision-making. This renders previous evidence synthesis efforts less valuable and may delay the implementation of evidence-based interventions.

The present findings raise an important practical question: how should behaviour analysts approach evidence-based decision-making when the available systematic reviews are overwhelmingly rated as critically low? A critically low AMSTAR 2 rating does not imply that a review is without value, but rather that there are substantial limitations in the methodological features underpinning its conclusions. In the absence of higher-quality syntheses, existing reviews may still serve as useful starting points for identifying relevant interventions and providing updated summaries of the literature. Practitioners should interpret their conclusions cautiously, however, and where feasible, decision-making may be strengthened by consulting primary studies directly, examining the methodological features of included research, and triangulating across multiple sources rather than relying on a single review. At a systems level, these findings underscore the need for professional organisations, training programs, and journals to prioritise improvements in evidence synthesis practices so that clinicians are better supported in making informed decisions.

Despite the overall low-quality ratings, we observed some patterns of high adherence to specific AMSTAR 2 items that represent notable methodological strengths within behaviour analysis systematic reviews. A high proportion of studies employed independent raters for data extraction (80%) and study selection (65%), demonstrating awareness of the importance of reducing reviewer bias and enhancing reliability. The use of multiple independent reviewers is widely recognised as best practice, as it minimises the risk of subjective interpretation. These adherence rates are consistent with other umbrella reviews in adjacent fields that have reported relatively higher adherence to these items compared to other AMSTAR 2 criteria [52,53].

We undertook sensitivity analyses to understand whether the average improvement in R-AMSTAR scores was primarily driven by a small number of early reviews. Overall, this analysis suggested that while temporal improvement in adherence spans the entire study period, its magnitude may have been influenced by the rapid adoption of methodological norms in earlier years. Excluding reviews published before 2010 attenuated the average marginal effect (from 1.08% in the primary model to 0.7% per year), suggesting that early studies contributed a steeper rate of initial change. This pattern is consistent with the rapid methodological development that followed the widespread adoption of reporting standards such as PRISMA in 2009, which may have produced a period of accelerated improvement as the field oriented itself around emerging best practices. Nevertheless, the persistence of a positive trend in the post-2010 data confirms that progress is not merely an artifact of early outliers but reflects a consistent, ongoing refinement of systematic review quality.

We also examined whether methodological quality differed between reviews that included a meta-analysis and those that did not. This revealed that meta-analytic reviews tended to score modestly higher using the R-AMSTAR tool than those without a meta-analysis. The finding may reflect meaningful differences in researcher expertise and methodological rigour. Conducting a meta-analysis requires familiarity with quantitative synthesis methods, heterogeneity assessment, and publication bias evaluation. These are competencies that overlap substantially with the broader methodological standards captured by appraisal tools like AMSTAR 2. Researchers undertaking meta-analyses may therefore be more conversant with systematic review methodology than those producing narrative syntheses. This interpretation is consistent with the concern that the term ‘systematic review’ is being used to describe reviews conducted without the procedural rigour that the label implies. If a proportion of non-meta-analytic reviews in our sample represent such cases, lower average methodological quality scores might be expected. Only 16 reviews in our sample conducted a meta-analysis, so the analysis should be interpreted with caution.

One limitation of our review is the applicability of AMSTAR 2 to behaviour analysis research traditions, particularly given the prominence of single-case experimental designs. Some AMSTAR 2 criteria, such as explicit PICO-framing or certain meta-analytic assumptions derived from RCT traditions, may align less naturally with single-case research. Only 8 of 64 studies fully met the requirements for establishing a clear PICO (Population, Intervention, Comparison, Outcome) framework. Consistent with our findings, Jamshidi et al. [21] found that only 1% of studies developed the research question in the PICO format. Single-case experimental designs can (although not always) lack a traditional “comparison” group or condition, whereby participants can sometimes serve as their own controls across experimental phases. Furthermore, many of the reviews in our sample were broad procedural examinations rather than investigations of defined or unified interventions for specific populations. For example, Page et al. [54] explored the general effectiveness of self-monitoring and technology to increase physical activity. Moreover, behaviour analytic studies often employ what Johnson and Cook [55] describe as a dynamic-inductive approach (i.e., exploratory) to research, rather than a deductive-static (i.e., confirmatory) approach. In practice, this means that interventions within the behaviour analytic research tradition are adapted or changed during the course of a study, based on the effects that are observed. This makes it difficult to define specific interventions a priori in the manner expected by the PICO framework.

It is possible, therefore, that a portion of the low ratings reflects imperfect alignment between AMSTAR 2 and the epistemological foundations of behaviour analysis. Given that AMSTAR 2 was developed within health intervention research traditions, some methodological mismatch is perhaps unsurprising. However, in our view it would be a mistake to attribute the observed deficiencies solely to this misalignment. Many of the most consistently missed criteria in our sample, including protocol registration, transparent justification of exclusions, risk of bias assessment, and evaluation of publication bias, are broadly applicable safeguards against bias, not requirements specific to RCT-based health intervention reviews. Future work may explore whether adaptations of existing appraisal tools like AMSTAR 2 are warranted for single-case research traditions, while retaining core commitments to transparency, reproducibility, and bias minimisation.

These methodological characteristics also raise broader concerns about the compatibility between single-case research design principles and traditional evidence synthesis approaches. A characteristic of single-case research designs as used in behaviour analysis is the demonstration of functional relations. Arguably, this ideal may inadvertently promote publication bias through multiple pathways. At the individual study level, the procedures or interventions may be modified until they demonstrate ‘effectiveness’, making it unlikely that unsuccessful interventions will appear in the final published record. At the publication level, studies may be less likely to be written up and submitted for peer review unless they ‘work’. Beyond these two pathways, there is a further concern at the level of outcome reporting: even within studies that are written up and published, authors may include phases or participants that demonstrate the clearest effects, while downplaying or omitting those that do not. Taken together, these pathways create conditions in which the published literature may systematically overrepresent positive findings at multiple levels simultaneously. This is not a trivial concern. If the pool of studies available for synthesis is unrepresentative of the true distribution of intervention effects, then meta-analytic estimates may be substantially inflated, regardless of how rigorously the review itself was conducted. We regard this as a serious and as yet unresolved issue for the field, and one that deserves dedicated empirical and methodological attention. We reiterate recent calls for behaviour analysts to engage with methods that can ascertain the nature and extent of publication bias in the literature [56], and suggest that until greater clarity is achieved, meta-analytic conclusions drawn from this literature should be interpreted with considerable caution.

A limitation of the present review concerns the scope of journals included in the search strategy. Although the seven behaviour-analytic journals represent key outlets for behaviour-analytic scholarship, relevant systematic reviews and meta-analyses on behaviour-analytic interventions are also often published in broader psychological and educational journals. Our findings should therefore be interpreted as reflecting the state of synthesised evidence within outlets that exclusively publish behaviour-analytic research, rather than the entirety of behaviour-analytic scholarship. It is possible that behaviour-analytic research published in more generalist outlets may be subject to editorial policies, reporting standards, and methodological expectations that differ from those characteristic of behaviour analysis-specific journals, including practices that are aligned with contemporary standards for systematic review conduct and transparency. Future research would benefit from extending search strategies to include behaviour analytic reviews that have been published in mainstream psychological and educational journals in order to provide a more comprehensive characterisation of the evidence base and to examine differences in reporting practices across publication contexts.

A further limitation of our approach was the inclusion of systematic reviews that examined non-intervention or basic research topics [34,36]. Arguably, restricting our analysis to reviews that specifically evaluated the effectiveness of interventions might have provided a more focused assessment of methodological quality in clinical evidence synthesis. However, we maintained the approach specified in our preregistered protocol to ensure transparency and avoid post-hoc modifications to our methodology. To address this concern, we conducted a sensitivity analysis including only those studies that evaluated interventions or procedures related to socially significant outcomes. The results of this restricted analysis were similar to our main findings, suggesting that the inclusion of non-intervention reviews did not substantially impact our conclusions regarding systematic review quality in behaviour analysis. Additionally, we had initially planned to examine whether reviews focused on neurodivergent populations were associated with differences in methodological adherence. This analysis was not conducted, however, as we identified only a small number of studies in which participants were exclusively from these populations (11 studies).

It is standard practice in umbrella reviews to assess citation overlap among included reviews, typically via a citation overlap matrix. This approach addresses the concern that multiple included reviews may draw on the same primary studies, such that treating those reviews as independent sources of evidence could inflate confidence in shared conclusions. We did not include a citation overlap matrix here. Our unit of analysis was the review itself; overlap in the primary studies drawn upon by these reviews was incidental to our analysis. Reporting overlap could even mislead readers by implying that our conclusions depend on the independence of the underlying primary literature, when they do not.

Our findings have implications for the authors of systematic reviews in behaviour analysis and those who shape research standards within the field. We highlight two priorities that, if adopted consistently, would address many of the most prevalent methodological shortcomings identified here. First, review protocols should be registered prospectively on publicly accessible platforms such as PROSPERO or the Open Science Framework. Protocol registration is a low-cost strategy that strengthens transparency, reduces the risk of selective reporting, and safeguards against post hoc modification of research questions or analysis plans. Second, authors should adhere closely to established reporting guidelines such as PRISMA, and in a way that is substantive rather than procedural. Many of the limitations identified in the reviews are directly addressed in PRISMA, including comprehensive search strategies, transparent documentation of exclusion decisions, risk of bias assessment, and evaluation of publication bias. The present findings suggest a gap between stated compliance and their meaningful application. Journal editors and reviewers are well placed to ensure that reporting guidelines are applied substantively rather than cited as a formality.

In conclusion, our evaluation of methodological quality in behaviour analysis systematic reviews reveals both significant challenges and emerging opportunities for the field. While the universal classification of reviews as ‘critically low’ quality according to AMSTAR 2 criteria highlights substantial gaps between current practices and established methodological standards, the consistent improvement in adherence over time suggests capacity for positive change. Moving forward, we recommend that the behaviour analysis community engage in coordinated efforts involving researchers, journal editors, reviewers, and professional organisations to establish and enforce methodological standards that balance the unique characteristics of behaviour analytic research with the need for transparent, rigorous evidence synthesis. Without these efforts, the field risks perpetuating a cycle of weak evidence synthesis that undermines the translation of research into practice for the populations we serve.

## Supporting information

S1 File s1_protocol_changes; Protocol changes. (DOCX)

S2 File s2_study_list; Full list of included and excluded studies. (DOCX)

S3 File s3_study_details; Descriptive results of included studies. (DOCX)

S4 File s4_amstar_scores; Study-level AMSTAR-2 results. (DOCX)

S5 File s5_ramstar_scores; Study-level R-AMSTAR results. (DOCX)

S6 File s6_PRISMA_2020_abstract_checklist. (DOC)

S7 File s7_PRISMA_2020_checklist. (DOC)

S8 File s8_search. (DOCX)
