# Supplementary material for: Evaluating the quality of systematic reviews and meta-analyses published in behaviour analysis journals: An umbrella review
Source: PLoS One. 2026 Jun 26;21(6):e0350142. doi: 10.1371/journal.pone.0350142 (PMC13309035; doi:10.1371/journal.pone.0350142)
Supplement: S1 File — (DOCX) [file pone.0350142.s001.docx]

**Summary of Protocol Changes**

**Initial preregistration:** [**https://doi.org/10.17605/OSF.IO/DMK4W**](https://doi.org/10.17605/OSF.IO/DMK4W)

**Updated preregistration:** [**https://doi.org/10.17605/OSF.IO/U38Z4**](https://doi.org/10.17605/OSF.IO/U38Z4)

**The following changes were made to the initial preregistration:**

**Sampling frame narrowed:** The original protocol planned to search a broad set of 19 journals selected via three criteria (ABAI/SEAB outlets; journals indexed in King et al., 2020 that explicitly reference behaviour analysis; plus titles suggested by an external expert panel). Following preliminary searching and screening it was evident that this generated a volume of papers that was beyond the resource capacity of the research team. The updated protocol **reduced the scope to seven flagship ABAI/SEAB journals** to align with resources and practitioner relevance.

**Databases clarified:** The original listed **PsycInfo and ProQuest** in general terms; the update specifies **ProQuest-hosted APA PsycArticles, APA PsycInfo, and ASSIA.**

**Study types refined:** The update **explicitly excludes scoping reviews**, whereas the original did not list this exclusion, tightening eligibility around SRs/MAs.

**Aims adjusted:** The original included an aim to test whether quality (AMSTAR-2/R-AMSTAR) **varied by publication outlet**; the updated protocol **removed this outlet-predictor aim**, focusing on overall quality and temporal change. Because many publication types were represented by very few papers within individual journals, and we were expecting a change in quality over time, the team felt a comparative analysis at the level of individual journal would be minimally informative.

**Subgroup analysis added:** The updated protocol introduced a **population subgroup analysis** (autistic individuals and those with intellectual and developmental disabilities vs other) this analysis was **not specified** in the original.

**Data extraction expanded:** The update adds an explicit field noting whether a **meta-analysis was conducted (yes/no)** for each review; however, this was implicitly part of the AMSTAR-2 scoring anyway.
