# Supplementary material for: Evaluating the quality of systematic reviews and meta-analyses published in behaviour analysis journals: An umbrella review
Source: PLoS One. 2026 Jun 26;21(6):e0350142. doi: 10.1371/journal.pone.0350142 (PMC13309035; doi:10.1371/journal.pone.0350142)
Supplement: S2 File — (DOCX) [file pone.0350142.s002.docx]

**List of Included and Excluded Studies**

The following list contains the 90 studies that were subjected to screening following searches. Those studies (26 in total) marked with a * were *excluded* at the study screening phase. All remaining studies (64 in total) were included in the analysis.

*1. Aydin O, Yassikaya MY. Validity and Reliability Analysis of the PlotDigitizer Software Program for Data Extraction from Single-Case Graphs. Perspect Behav Sci. 2022;45: 239–257. doi:10.1007/s40614-021-00284-0

2. Bal A, Perzigian ABT. Evidence-based Interventions for Immigrant Students Experiencing Behavioral and Academic Problems: A Systematic Review of the Literature. Education and Treatment of Children. 2013;36: 5–28. doi:10.1353/etc.2013.0044

3. Barrett K, O’Connor M, McHugh L. A Systematic Review of Values-Based Psychometric Tools Within Acceptance and Commitment Therapy (ACT). Psychol Rec. 2019;69: 457–485. doi:10.1007/s40732-019-00352-7

*4. Barrett PM, Cooper M, Stallard P, Zeggio L, Gallegos-Guajardo J. Effective Evaluation of the FRIENDS Anxiety Prevention Program in School Settings: A Response to Maggin and Johnson. Education and Treatment of Children. 2017;40: 97–110. doi:10.1353/etc.2017.0006

5. Beck C, Garcia Y, Brothers L, Mahoney A, Rancourt RC, Andrews M. A Systematic Review of the Impact of Derived Relational Responding Technology in Raising Intelligence Scores. Psychol Rec. 2023;73: 339–361. doi:10.1007/s40732-023-00546-0

*6. Becraft JL, Borrero JC, Sun S, McKenzie AA. A primer for using multilevel models to meta‐analyze single case design data with AB phases. J of App Behav Analysis. 2020;53: 1799–1821. doi:10.1002/jaba.698

7. Boyle MA, Adamson RM. Systematic Review of Functional Analysis and Treatment of Elopement (2000–2015). Behav Analysis Practice. 2017;10: 375–385. doi:10.1007/s40617-017-0191-y

8. Bouck EC, Park J. A Systematic Review of the Literature on Mathematics Manipulatives to Support Students with Disabilities. Education and Treatment of Children. 2018;41: 65–106. doi:10.1353/etc.2018.0003

9. Bowman-Perrott L, deMarín S, Mahadevan L, Etchells M. Assessing the Academic, Social, and Language Production Outcomes of English Language Learners Engaged in Peer Tutoring: A Systematic Review. Education and Treatment of Children. 2016;39: 359–388. doi:10.1353/etc.2016.0016

10. Brodsky J, Fienup DM. Sidman Goes to College: A Meta-Analysis of Equivalence-Based Instruction in Higher Education. Perspect Behav Sci. 2018;41: 95–119. doi:10.1007/s40614-018-0150-0

11. Brown C, Maggin DM, Buren M. Systematic Review of Cultural Adaptations of School-based Social, Emotional, and Behavioral Interventions for Students of Color. Education and Treatment of Children. 2018;41: 431–456. doi:10.1353/etc.2018.0024

12. Cameron J, Banko KM, Pierce WD. Pervasive negative effects of rewards on intrinsic motivation: The myth continues. Behav Analyst. 2001;24: 1–44. doi:10.1007/BF03392017

13. Carvalho AAS, Mizael TM, Sampaio AAS. Racial Prejudice and Police Stops: A Systematic Review of the Empirical Literature. Behav Analysis Practice. 2022;15: 1213–1220. doi:10.1007/s40617-021-00578-4

*14. Colom R, Contreras MJ, Arend I, Leal OG, Santacreu J. Sex Differences in Verbal Reasoning are Mediated by Sex Differences in Spatial Ability. Psychol Rec. 2004;54: 365–372. doi:10.1007/BF03395479

15. Contreras BP, Tate SA, Morris SL, Kahng S. A systematic review of the correspondence between descriptive assessment and functional analysis. J of App Behav Analysis. 2023;56: 146–165. doi:10.1002/jaba.958

*16. Criollo AB, Díaz-Muelle S, Ruiz FJ, García-Martín MB. Common Physical Properties Improve Metaphor Effect Even in the Context of Multiple Examples. Psychol Rec. 2018;68: 513–523. doi:10.1007/s40732-018-0297-9

*17. Derenne A, Baron A. Human sensitivity to reinforcement: A comment on Kollins, Newland, and Critchfield’s (1997) quantitative literature review. Behav Analysis. 1999;22: 35–41. doi:10.1007/BF03391976

18. DeSouza AA, Akers JS, Fisher WW. Empirical Application of Skinner’s Verbal Behavior to Interventions for Children with Autism: A Review. Analysis Verbal Behav. 2017;33: 229–259. doi:10.1007/s40616-017-0093-7

*19. Dowdy A, Hantula DA, Travers JC, Tincani M. Meta-Analytic Methods to Detect Publication Bias in Behavior Science Research. Perspect Behav Sci. 2022;45: 37–52. doi:10.1007/s40614-021-00303-0

20. Dowdy A, Jessel J, Saini V, Peltier C. Structured visual analysis of single‐case experimental design data: Developments and technological advancements. J of App Behav Analysis. 2022;55: 451–462. doi:10.1002/jaba.899

*21. Dowdy A, Peltier C, Tincani M, Schneider WJ, Hantula DA, Travers JC. Meta‐analyses and effect sizes in applied behavior analysis: A review and discussion. J of App Behav Analysis. 2021;54: 1317–1340. doi:10.1002/jaba.862

22. Dowdy A, Tincani M, Schneider WJ. Evaluation of publication bias in response interruption and redirection: A meta‐analysis. J of App Behav Analysis. 2020;53: 2151–2171. doi:10.1002/jaba.724

23. Dunn ME, Shelnut J, Ryan JB, Katsiyannis A. A Systematic Review of Peer-Mediated Interventions on the Academic Achievement of Students with Emotional/Behavioral Disorders. Education and Treatment of Children. 2017;40: 497–524. doi:10.1353/etc.2017.0022

24. Ennis RP, Royer DJ, Lane KL, Griffith CE. A Systematic Review of Precorrection in PK-12 Settings. Education and Treatment of Children. 2017;40: 465–495. doi:10.1353/etc.2017.0021

25. Erion J. Parent tutoring: A meta-analysis. Education & Treatment of Children. 2006;29: 79–106.

*26. Foorman BR, Lee L, Smith K. Implementing Evidence-Based Reading Practices in K–3 Classrooms. Educ Treat Child. 2020;43: 49–55. doi:10.1007/s43494-020-00005-3

27. Fox RA, Leif ES, Moore DW, Furlonger B, Anderson A, Sharma U. A Systematic Review of the Facilitators and Barriers to the Sustained Implementation of School-Wide Positive Behavioral Interventions and Supports. Educ Treat Child. 2022;45: 105–126. doi:10.1007/s43494-021-00056-0

*28. Fox RA, Leif ES, Moore DW, Furlonger B, Anderson A, Sharma U. Correction to: A Systematic Review of the Facilitators and Barriers to the Sustained Implementation of School-Wide Positive Behavioral Interventions and Supports. Educ Treat Child. 2022; 127. doi:10.1007/s43494-021-00062-2

29. Frampton SE, Munk GT, Shillingsburg LA, Shillingsburg MA. A Systematic Review and Quality Appraisal of Applications of Direct Instruction with Children with Autism Spectrum Disorder. Perspect Behav Sci. 2021;44: 245–266. doi:10.1007/s40614-021-00292-0

30. Gardner AW, Spencer TD, Boelter EW, DuBard M, Jennett HK. A Systematic Review of Brief Functional Analysis Methodology with Typically Developing Children. Education and Treatment of Children. 2012;35: 313–332. doi:10.1353/etc.2012.0014

31. Germansky S, Reichow B, Martin M, Snyder P. A Systematic Review of Caregiver-Implemented Functional Analyses. Behav Analysis Practice. 2020;13: 698–713. doi:10.1007/s40617-019-00404-y

*32. Gresham FM. Social competence and students with behavior disorders: Where we’ve been, where we are, and where we should go. Education and Treatment of Children. 1997;20: 233-249.

33. Groves EA, Najafichaghabouri M, Seel CJ, Fischer S, Thomas C, Joslyn PR. A Systematic Review of Group Contingencies in Alternative Education Settings. Educ Treat Child. 2023;46: 145–164. doi:10.1007/s43494-023-00095-9

34. Haddock JN, Hagopian LP. Competing stimulus assessments: A systematic review. J of App Behav Analysis. 2020;53: 1982–2001. doi:10.1002/jaba.754

*35. Harte C, Barnes-Holmes D. The Status of Rule-Governed Behavior as Pliance, Tracking and Augmenting within Relational Frame Theory: Middle-Level Rather than Technical Terms. Psychol Rec. 2022;72: 145–158. doi:10.1007/s40732-021-00458-x

36. Hawken LS, Bundock K, Kladis K, O’Keeffe B, Barrett CA. Systematic Review of the Check-in, Check-out Intervention for Students At Risk for Emotional and Behavioral Disorders. Education and Treatment of Children. 2014;37: 635–658. doi:10.1353/etc.2014.0030

37. Heinicke MR, Carr JE, Copsey CJ. Assessing preferences of individuals with developmental disabilities using alternative stimulus modalities: A systematic review. J of App Behav Analysis. 2019;52: 847–869. doi:10.1002/jaba.565

*38. Higgins ST, Kurti AN, Davis DR. Voucher-Based Contingency Management is Efficacious but Underutilized in Treating Addictions. Perspect Behav Sci. 2019;42: 501–524. doi:10.1007/s40614-019-00216-z

39. Hirsch SE, Randall K, Bradshaw C, Lloyd JW. Professional Learning and Development in Classroom Management for Novice Teachers: A Systematic Review. Educ Treat Child. 2021;44: 291–307. doi:10.1007/s43494-021-00042-6

40. Hurd AM, Nercesian SJ, Brown KR, Visser EJ. A Systematic Review on Functional Analysis of Noncompliance. Educ Treat Child. 2023;46: 45–58. doi:10.1007/s43494-023-00091-z

41. Jaehnig W, Miller ML. Feedback Types in Programmed Instruction: A Systematic Review. Psychol Rec. 2007;57: 219–232. doi:10.1007/BF03395573

42. Kestner KM, Finch KR, Kolb RL. Systematic Review of Procedures and Outcomes of Choice-Based Interventions with Children. Educ Treat Child. 2023;46: 77–106. doi:10.1007/s43494-023-00088-8

43. King S, Kennedy K, Powelson A. Behavior Management Interventions for School Buses: A Systematic Review. Education and Treatment of Children. 2019;42: 101–128. doi:10.1353/etc.2019.0005

*44. King SA, Kostewicz D, Enders O, Burch T, Chitiyo A, Taylor J, et al. Search and Selection Procedures of Literature Reviews in Behavior Analysis. Perspect Behav Sci. 2020;43: 725–760. doi:10.1007/s40614-020-00265-9

*45. Kinney C, Weatherly N, Burns G, Nicholson K. Improving Visual Inspection, Interrater Agreement, and Standardization with the Graphic Variability Quotient. Psychol Rec. 2023;73: 75–96. doi:10.1007/s40732-022-00522-0

*46. Kollins SH, Newland MC, Critchfield TS. Quantitative integration of single-subject studies: Methods and misinterpretations. Behav Analyst. 1999;22: 149–157. doi:10.1007/BF03391992

47. Konrad M, Joseph LM, Eveleigh E. A Meta-Analytic Review of Guided Notes. Education and Treatment of Children. 2009;32: 421–444. doi:10.1353/etc.0.0066

48. Kranak MP, Andzik NR, Jones C, Hall H. A Systematic Review of Supervision Research Related to Board Certified Behavior Analysts. Behav Analysis Practice. 2023;16: 1006–1021. doi:10.1007/s40617-023-00805-0

49. Kupzyk S, LaBrot ZC, Collins MJ. An Updated Systematic Review on Parent Tutoring. Educ Treat Child. 2023;46: 59–75. doi:10.1007/s43494-023-00090-0

*50. LaMarca V, LaMarca J. Designing Receptive Language Programs: Pushing the Boundaries of Research and Practice. Behav Analysis Practice. 2018;11: 479–495. doi:10.1007/s40617-018-0208-1

*51. Larzelere RE, Daly, DL, Davis, JL, Chmelka MB, Handwerk ML. Outcome Evaluation of Girls and Boys Town’s Family Home Program. Education and Treatment of Children . 2004;27: 130-149.

*52. Lattal KA, Kuroda T, Cook JE. Early extinction effects following intermittent reinforcement: Little evidence of extinction bursts. J Exper Analysis Behavior. 2020;114: 47–59. doi:10.1002/jeab.616

53. Losinski M, Sanders S, Katsiyannis A, Wiseman N. A Meta-Analysis of Interventions to Improve the Compliance of Students with Disabilities. Education and Treatment of Children. 2017;40: 435–463. doi:10.1353/etc.2017.0020

54. MacSuga-Gage AS, Simonsen B. Examining the Effects of Teacher-Directed Opportunities to Respond on Student Outcomes: A Systematic Review of the Literature. Education and Treatment of Children. 2015;38: 211–239. doi:10.1353/etc.2015.0009

55. Maggin DM, Johnson AH. A Meta-Analytic Evaluation of the FRIENDS Program for Preventing Anxiety in Student Populations. Education and Treatment of Children. 2014;37: 277–306. doi:10.1353/etc.2014.0018

*56. Maggin DM, Johnson AH. Meta-Analytic Evaluation of FRIENDS: A Rejoinder of. Education and Treatment of Children. 2019;42: 129–146. doi:10.1353/etc.2019.0006

*57. Mason L, Otero M. Just How Effective is Direct Instruction? Perspect Behav Sci. 2021;44: 225–244. doi:10.1007/s40614-021-00295-x

58. McCormack JC, Elliffe D, Virués‐Ortega J. Quantifying the effects of the differential outcomes procedure in humans: A systematic review and a meta‐analysis. J of App Behav Analysis. 2019;52: 870–892. doi:10.1002/jaba.578

59. McCoy A, McNaughton D. Training Education Professionals to Use the Picture Exchange Communication System: a Review of the Literature. Behav Analysis Practice. 2019;12: 667–676. doi:10.1007/s40617-018-00296-4

60. Nemer SL, Sutherland KS, Chow JC, Kunemund RL. A Systematic Literature Review Identifying Dimensions of Teacher Attributions for Challenging Student Behavior. Education and Treatment of Children. 2019;42: 557–578. doi:10.1353/etc.2019.0026

61. Nesselrode R, Falcomata TS, Hills L, Erhard P. Functional Analysis in Public School Settings: A Systematic Review of the Literature. Behav Analysis Practice. 2022;15: 958–970. doi:10.1007/s40617-022-00679-8

*62. Oakes WP, Mathur SR, Clark HG, Common EA. Introduction to the Special Issue: Teacher Educators for Children With Behavior Disorders (TECBD) Conference on Severe Behavior Disorders of Children and Youth. Education and Treatment of Children. 2017;40: 429–433. doi:10.1353/etc.2017.0019

63. Odum AL, Becker RJ, Haynes JM, Galizio A, Frye CCJ, Downey H, et al. Delay discounting of different outcomes: Review and theory. J Exper Analysis Behavior. 2020;113: 657–679. doi:10.1002/jeab.589

*64. O’Keeffe BV, Slocum TA, Burlingame C, Snyder K, Bundock K. Comparing Results of Systematic Reviews: Parallel Reviews of Research on Repeated Reading. Education and Treatment of Children. 2012;35: 333–366. doi:10.1353/etc.2012.0006

65. Page EJ, Massey AS, Prado-Romero PN, Albadawi S. The Use of Self-Monitoring and Technology to Increase Physical Activity: A Review of the Literature. Perspect Behav Sci. 2020;43: 501–514. doi:10.1007/s40614-020-00260-0

66. Park E-Y, Blair K-SC. Check-in/Check-out Implementation in Schools: a Meta-Analysis of Group Design Studies. Educ Treat Child. 2020;43: 361–375. doi:10.1007/s43494-020-00030-2

67. Perrin J, Morris C, Kestner K. Resurgence of Clinically Relevant Behavior: A Systematic Review. Educ Treat Child. 2022;45: 85–104. doi:10.1007/s43494-021-00054-2

68. Podlesnik CA, Ritchey CM, Waits J, Gilroy SP. A Comprehensive Systematic Review of Procedures and Analyses Used in Basic and Preclinical Studies of Resurgence, 1970–2020. Perspect Behav Sci. 2023;46: 137–184. doi:10.1007/s40614-022-00361-y

69. Rajaraman A, Hanley GP. Mand compliance as a contingency controlling problem behavior: A systematic review. J of App Behav Analysis. 2021;54: 103–121. doi:10.1002/jaba.758

70. Regnier SD, Traxler HK, Devoto A, DeFulio A. A Systematic Review of Treatment Maintenance Strategies in Token Economies: Implications for Contingency Management. Perspect Behav Sci. 2022;45: 819–861. doi:10.1007/s40614-022-00358-7

71. Richman DM, Barnard‐Brak L, Grubb L, Bosch A, Abby L. Meta‐analysis of noncontingent reinforcement effects on problem behavior. J of App Behav Analysis. 2015;48: 131–152. doi:10.1002/jaba.189

72. Riden BS, Snyder SM, Fowkes CL, Yuschak AE. Using Eye Gaze Preference Assessments to Identify Preferred Stimuli: A Systematic Review. Educ Treat Child. 2022;45: 305–320. doi:10.1007/s43494-022-00076-4

73. Rubio EK, McMahon MXH, Volkert VM. A systematic review of physical guidance procedures as an open‐mouth prompt to increase acceptance for children with pediatric feeding disorders. J of App Behav Analysis. 2021;54: 144–167. doi:10.1002/jaba.782

74. Saini V, Kadey HJ, Paszek KJ, Roane HS. A systematic review of functional analysis in pediatric feeding disorders. J of App Behav Analysis. 2019;52: 1161–1175. doi:10.1002/jaba.637

75. Saini V, Mitteer DR. A review of investigations of operant renewal with human participants: Implications for theory and practice. J Exper Analysis Behavior. 2020;113: 105–123. doi:10.1002/jeab.562

76. Sivaraman M, Barnes-Holmes D. Naming: What Do We Know So Far? A Systematic Review. Perspect Behav Sci. 2023;46: 585–615. doi:10.1007/s40614-023-00374-1

77. Sivaraman M, Fahmie TA. A systematic review of cultural adaptations in the global application of ABA ‐based telehealth services. J of App Behav Analysis. 2020;53: 1838–1855. doi:10.1002/jaba.763

*78. Slocum TA, Detrich R, Spencer TD. Evaluating the Validity of Systematic Reviews to Identify Empirically Supported Treatments. Education and Treatment of Children. 2012;35: 201–233. doi:10.1353/etc.2012.0009

79. Stinson L, Liu Y, Dallery J. Ecological Momentary Assessment: A Systematic Review of Validity Research. Perspect Behav Sci. 2022;45: 469–493. doi:10.1007/s40614-022-00339-w

*80. Stockard J. Building a More Effective, Equitable, and Compassionate Educational System: The Role of Direct Instruction. Perspect Behav Sci. 2021;44: 147–167. doi:10.1007/s40614-021-00287-x

81. Suarez VD, Moon EI, Najdowski AC. Systematic Review of Acceptance and Commitment Training Components in the Behavioral Intervention of Individuals with Autism and Developmental Disorders. Behav Analysis Practice. 2022;15: 126–140. doi:10.1007/s40617-021-00567-7

82. Sweigart CA, Collins LW, Evanovich LL, Cook SC. An Evaluation of the Evidence Base for Performance Feedback to Improve Teacher Praise Using CEC’s Quality Indicators. Education and Treatment of Children. 2016;39: 419–444. doi:10.1353/etc.2016.0019

83. Thoele JM, DeAngelo S. An Examination of Social Validity for Students with Emotional Behavioral Disorders: Has Progress Been Made? Educ Treat Child. 2023;46: 279–302. doi:10.1007/s43494-023-00109-6

84. Tincani M, Miller J, Lorah ER, Nepo K. Systematic Review of Verbal Operants in Speech Generating Device Research from Skinner’s Analysis of Verbal Behavior. Perspect Behav Sci. 2020;43: 387–413. doi:10.1007/s40614-020-00243-1

85. Trevor M, Park E-Y, Blair K-SC. A Meta-Analysis of Safety Skills Interventions for Individuals with Intellectual Disabilities. Educ Treat Child. 2021;44: 309–331. doi:10.1007/s43494-021-00051-5

*86. Vlaeyen JWS, Wicksell RK, Simons LE, Gentili C, De TK, Tate RL, et al. From Boulder to Stockholm in 70 Years: Single Case Experimental Designs in Clinical Research. Psychol Rec. 2020;70: 659–670. doi:10.1007/s40732-020-00402-5

87. Weinsztok SC, Goldman KJ, DeLeon IG. Assessing Parameters of Reinforcement on Efficiency of Acquisition: A Systematic Review. Behav Analysis Practice. 2023;16: 76–92. doi:10.1007/s40617-022-00715-7

88. White WA. A meta-analysis of the effects of Direct Instruction in special education. . Education and Treatment of Children. 1988;11: 364–374.

89. Wong T, Falcomata TS, Barnett M. The Collateral Effects of Antecedent Exercise on Stereotypy and Other Nonstereotypic Behaviors Exhibited by Individuals with Autism Spectrum Disorder: A Systematic Review. Behav Analysis Practice. 2023;16: 407–420. doi:10.1007/s40617-022-00746-0

90. Wooderson JR, Bizo LA, Young K. A Systematic Review of Emergent Learning Outcomes Produced by Foreign language Tact Training. Analysis Verbal Behav. 2022;38: 157–178. doi:10.1007/s40616-022-00170-z
