# Supplementary material for: Evaluating the quality of systematic reviews and meta-analyses published in behaviour analysis journals: An umbrella review
Source: PLoS One. 2026 Jun 26;21(6):e0350142. doi: 10.1371/journal.pone.0350142 (PMC13309035; doi:10.1371/journal.pone.0350142)
Supplement: S3 File — (DOCX) [file pone.0350142.s003.docx]

| **Lead Author** | **Year** | **Outlet** | **Country** | **Intervention** | **Outcome** | **Studies** | **Design** | **Funding** | **Participants** | **PRISMA** | **Int** |
| --- | --- | --- | --- | --- | --- | --- | --- | --- | --- | --- | --- |
| Bal | 2013 | ETC | USA | Academic and behavioural interventions | Academic proficiency and behavioural outcomes | 6 | SCED; between group; pre-post only group | not reported | ANT, CNT, CND | no | yes |
| Barrett | 2019 | PR | Ireland | Values-based psychometric instrument | Reliability and validity | 21 | Correlational | not reported | OTH: Clinical and non-clinical sample | no | no |
| Beck | 2023 | PR | USA; Colombia | Derived relational responding procedures | Intelligence quotients | 15 | SCED, Between group, single group | not reported | ANT, CNT, CND | yes | yes |
| Bouck | 2018 | ETC | USA | Use of manipulatives to teach mathematics | Mathematical concepts | 36 | SCED, between group | not reported | CNT, CND | no | yes |
| Bowman-Perrott | 2016 | ETC | USA | Peer tutoring | Academic and linguistic outcomes; social validity | 17 | SCED, between group, 1 case study | not reported | CNT, CND | no | yes |
| Boyle | 2017 | BAP | USA | Functional analysis and "function-based treatment" | Elopement | 12 | SCED only (although not explicitly reported) | not reported | OTH: Neurodiverse (age not reported) | no | yes |
| Brodsky | 2018 | POBS | USA | Equivalence-based instruction | Relevant academic skills | 28 | SCED, between group | not reported | ANT | no | yes |
| Brown | 2018 | ETC | USA | Cultural adaptations to school based social, emotional, and behavioural interventions (no intervention). | Social, emotional and behavioural outcomes | 10 | RCT; quasi exp; pre-post group; case studies. | not reported | OTH: Children and adolescents with Emotional and Behavioural Disorder (EBD) or at risk for EBD | no | yes |
| Cameron | 2001 | POBS | Canada | Rewards | Intrinsic motivation (measured as free choice or self-reported measures of task interest) | 145 | SCED, between group | yes | OTH: Not reported | no | yes |
| Carvalho | 2022 | BAP | USA | Racial prejudice | Police stops | 16 | Mixed methods, survey, group design | yes | ANT | yes | no |
| Contreras | 2023 | JABA | USA | n/a | Correspondence between experimental functional analysis and descriptive assessments | 48 | SCED | not reported | OTH: Neurotypical and neurodiverse (age not reported) | yes | yes |
| DeSouza | 2017 | TAVB | USA | Verbal operant-based teaching | Verbal operants | 172 | SCED | not reported | CND | no | yes |
| Dowdy | 2020 | JABA | USA | Response interruption and redirection | Publication bias | 38 | SCED | not reported | AND, CND | no | no |
| Dowdy | 2022 | JABA | Canada, USA | Occurrence of visual analysis in published papers | Frequency of structured visual analysis | 8 | SCED | not reported | NA | no | no |
| Dunn | 2017 | ETC | USA | Peer mediated interventions | Academic achievement; social validity | 24 | SCED, between group | yes | OTH: Children and adolescents “Emotionally Disturbed (ED), EBD, or Behaviourally Disordered (BD)” | yes | yes |
| Ennis | 2017 | ETC | USA | Pre-correction | Problem behaviour or on-task behaviour; social validity | 10 | SCED | not reported | CNT, CND | no | yes |
| Erion | 2006 | ETC | USA | Parent Tutoring | Academic skills | 37 | SCED, between group | yes | CNT | no | yes |
| Fox | 2022 | ETC | Australia; NZ | School-wide positive behaviour supports | Barriers and facilitators to implementation | 29 | Qualitative (interview; focus group); quantitative (survey). | yes | ANT | no | yes |
| Frampton | 2021 | POBS | USA | Direct Instruction | Intelligence quotients , language, reading | 16 | SCED, between group | not reported | CND | no | yes |
| Gardner | 2012 | ETC | USA | Brief functional analysis | Function of behaviour | 9 | SCED | not reported | CNT | no | yes |
| Germansky | 2020 | BAP | USA | Training caregivers to implement FAs | Conducting assessments | 36 | SCED | yes | ANT, CND | yes | yes |
| Groves | 2023 | ETC | Wales; USA | Group contingencies | On/off task; problem behaviour, disruptive behaviour; academic engagement; verbal responses of students; social validity | 21 | SCED | not reported | CNT, CND | yes | yes |
| Haddock | 2020 | JABA | USA | Competing stimulus assessments | Problem behaviour | 15 | SCED | yes | AND, CND | yes | yes |
| Hawken | 2014 | ETC | USA | Check in/Check out | Academic engagement; problem behaviour; on/off task; points earned; social validity | 28 | SCED, between group. | not reported | CNT, AND, CND | no | yes |
| Heinicke | 2019 | JABA | USA | Alternative modality stimulus preference assessments | Approach responses | 32 | SCED | not reported | AND, CND | yes | yes |
| Hirsch | 2021 | ETC | USA | Practice-based professional development | Teacher behaviour; student behaviour. | 8 | SCED, between group | not reported | ANT, CNT, OTH: Not reported | no | yes |
| Hurd | 2023 | ETC | USA | Functional analysis of non-compliance and subsequent antecedent and consequent-based interventions. | Non-compliance | 12 | SCED | not reported | CNT, AND | yes | yes |
| Jaehnig | 2007 | PR | USA | Feedback type | Acquisition of skills in programmed instruction | 31 | Not reported | not reported | OTH: Not reported | no | yes |
| Kestner | 2023 | ETC | USA | Choice-based interventions (Differential reinforcement and building choice in daily contexts). | Challenging behaviour; adherence to demands; task completion; self- control choices | 32 | SCED | not reported | CNT, CND | yes | yes |
| King | 2019 | ETC | USA | Driver training and use of operant procedures | Student behaviour on school bus | 18 | SCED, between group | not reported | CNT, CND, OTH: Not reported | no | yes |
| Konrad | 2009 | ETC | USA | Guided notes | Academic performance; note-taking; student engagement; social validity | 8 | SCED, cross sectional | not reported | ANT, CNT, CND | no | yes |
| Kranak | 2023 | BAP | USA | n/a | Supervision practices | 40 | SCED, survey | not reported | ANT | yes | no |
| Kupzyk | 2023 | ETC | USA | Parent tutoring | Academic performance | 30 | SCED & between group. | not reported | CNT, CND | yes | yes |
| Losinski | 2017 | ETC | USA | Various compliance-based interventions | Compliance | 28 | SCED | not reported | CNT, CND | no | yes |
| MacSuga-Gage | 2015 | ETC | USA | Teacher directed opportunities to respond | Student behaviour and academic outcomes | 15 | SCED, between group. | yes | CNT, CND | no | yes |
| Maggin | 2014 | ETC | USA | FRIENDS programme | Anxiety | 17 | Group (RCT, quasi) | not reported | OTH: Children; diagnosis not reported | no | yes |
| Maxfield | 2021 | ETC | USA, South Korea | Safety skills interventions | Safety skills | 31 | SCED | not reported | CND | yes | yes |
| McCormack | 2019 | JABA | New Zealand | Differential outcomes procedure | Acquisition of conditional discrimination accuracy, latency, error, trials to mastery | 35 | Between-group, Within-subject, SCED | not reported | ANT, CNT, AND, CND | no | yes |
| McCoy | 2019 | BAP | USA | Training educators to implement the Picture Exchange Communication System (PECS) | Educator performance in PECS | 7 | SCED, RCT, Non- Randomised Group | yes | ANT, AND, CND | no | yes |
| Nemer | 2019 | ETC | USA | None | Teacher-rated attributions | 25 | n/a | yes | ANT | no | no |
| Nesselrode | 2022 | BAP | USA | Functional analysis | Problem behaviour | 42 | SCED | none | OTH: Neurotypical and neurodiverse participants (age not reported) | no | yes |
| Odum | 2020 | JEAB | USA | Impact of outcome type | Delay discounting | 53 | not reported | not reported | OTH: Not reported | no | no |
| Page | 2020 | POBS | USA | Self-monitoring and technology | Physical activity | 20 | SCED | not reported | OTH: Children and adults; diagnosis not reported | no | yes |
| Park | 2020 | ETC | South Korea, USA | Check in/Check out | Problem behaviour, appropriate behaviour, social skills academic performance | 6 | Group (quasi, RCT) | none | CNT, CND | no | yes |
| Perrin | 2022 | ETC | USA | Schedules of reinforcement; schedule thinning; stimulus control; teaching alternative responses | Resurgence of clinically relevant behaviour; challenging behaviour | 22 | SCED | not reported | CNT, AND, CND | no | yes |
| Podlesnik | 2023 | POBS | USA | NA | Resurgence of behaviour | 200 | Between and within subject and combination of both. | not reported | ANT, CNT, AND, CND, NH | yes | no |
| Rajaraman | 2021 | JABA | USA | Mand compliance | Functional analysis characteristics, intervention characteristics | 22 | SCED | not reported | CND | yes | yes |
| Regnier | 2022 | POBS | USA | Token economies; treatment maintenance strategies (thinning or fading with transfer of control to natural reinforcers or self- management) | Treatment maintenance (various measures) | 37 | SCED and group (unclear re randomisation) | yes | ANT, CNT, AND, CND | no | yes |
| Richman | 2015 | JABA | USA | Noncontingent reinforcement | Problem behaviour | 55 | SCED | not reported | AND, CND | no | yes |
| Riden | 2022 | ETC | USA | Eye gaze preference assessment (no intervention) | Preference | 18 | not reported | not reported | ANT, CNT, AND, CND | yes | yes |
| Rubio | 2021 | JABA | USA | Physical guidance procedures | Food acceptance | 9 | SCED | not reported | CNT, CND | yes | yes |
| Saini | 2020 | JEAB | Canada, USA | NA | Operant renewal | 12 | SCED | not reported | ANT, CNT, AND, CND | yes | no |
| Saini | 2019 | JABA | Canada, USA | Functional analysis in feeding disorders | Function, participant and procedural characteristics, setting characteristics | 18 | SCED | not reported | CNT, CND | yes | no |
| Sivaraman | 2020 | JABA | Belgium, India, USA | Telehealth | Cultural adaptations to telehealth programmes | 9 | SCED and Group | not reported | ANT, CND | yes | yes |
| Sivaraman | 2023 | POBS | Wales; Northern Ireland | Various | Naming | 46 | Not reported | not reported | ANT, CNT, CND | yes | yes |
| Stinson | 2022 | POBS | USA | NA | Validity of Ecological Momentary Assessment | 32 | Cross sectional, correlational | not reported | ANT, CNT | yes | no |
| Suarez | 2022 | BAP | USA | Acceptance and Commitment Therapy components | Effects on behaviour | 29 | SCED | not reported | ANT, CND | yes | yes |
| Sweigart | 2016 | ETC | USA | Performance feedback. | Use of praise | 14 | SCED | not reported | ANT | no | yes |
| Thoele | 2023 | ETC | USA | NA | Social validity | 106 | SCED | none | CNT, OTH: EBD | no | no |
| Tincani | 2020 | POBS | USA | Speech-generating device interventions | Verbal operants | 56 | SCED, between group. | not reported | CNT, AND, CND | yes | yes |
| Weinsztok | 2023 | BAP | USA | Parameters of reinforcement within Discrete Trial Teaching | Acquisition efficiency | 34 | SCED | not reported | CND | yes | yes |
| White | 1988 | ETC | USA | Direct Instruction | Reading, mathematics, social, academic, spelling, health, writing, language | 25 | Group (RCT, quasi) | not reported | CND | no | yes |
| Wong | 2022 | BAP | USA | Antecedent exercise | Stereotypic and non-stereotypic behaviour | 14 | SCED and non-randomised group design (no control group) | not reported | AND, CND | no | yes |
| Wooderson | 2022 | TAVB | Australia | Tact training on emergent learning | Derived verbal behaviour | 10 | SCED | none | ANT, CNT | no | yes |

Note: BAP: Behavior Analysis in Practice; ETC: Education and Treatment of Children; JABA: Journal of Applied Behavior Analysis; JEAB: Journal of the Experimental Analysis of Behavior; POBS: Perspectives on Behavior Science; PR: Psychological Record; TAVB: The Analysis of Verbal Behavior; SCED: Single Case Experimental Design; RCT: Randomised Controlled Trial; ANT: Adult Neurotypical; CNT: Child Neurotypical; AND: Adult Neurodiverse (e.g., Adult with Autism Spectrum Disorder (ASD), Intellectual Disability (ID) or Developmental Disability (DD) (includes learning disability, Down Syndrome, or Pervasive Developmental Disorder); CND: Child Neurodiverse (e.g., Child with Autism Spectrum Disorder (ASD), Intellectual Disability (ID) or Developmental Disability (DD) (includes learning disability, Down Syndrome, or Pervasive Developmental Disorder); NH: Non-human; NA: Not applicable; OTH: Other; Int: Review of an intervention
