# Supplementary material for: Evaluating the quality of systematic reviews and meta-analyses published in behaviour analysis journals: An umbrella review
Source: PLoS One. 2026 Jun 26;21(6):e0350142. doi: 10.1371/journal.pone.0350142 (PMC13309035; doi:10.1371/journal.pone.0350142)
Supplement: S5 File — (DOCX) [file pone.0350142.s005.docx]

R-AMSTAR sub-item ratings by study

| **Study** | **1 a** | **1b** | **1 c** | **2 a** | **2b** | **2 c** | **2d** | **2 e** | **2 f** | **3 a** | **3b** | **3 c** | **3d** | **3 e** | **3 f** | **4 a** | **4b** | **4 c** | **4d** | **5 a** | **5b** | **5 c** | **5d** | **6 a** | **6b** | **6 c** | **7 a** | **7b** | **7 c** | **7d** | **7 e** | **8 a** | **8b** | **8 c** | **9 a** | **9b** | **9 c** | **9d** | **10a** | **10b** | **10c** | **11a** | **11b** | **11c** |
| --- | --- | --- | --- | --- | --- | --- | --- | --- | --- | --- | --- | --- | --- | --- | --- | --- | --- | --- | --- | --- | --- | --- | --- | --- | --- | --- | --- | --- | --- | --- | --- | --- | --- | --- | --- | --- | --- | --- | --- | --- | --- | --- | --- | --- |
| Bal (2023) | N | N | N | N | N | N | N | N | N | Y | Y | Y | Y | Y | N | N | Y | N | N | Y | N | N | N | Y | N | Y | N | N | Y | N | Y | Y | Y | Y | N | — | — | — | N | N | N | N | N | N |
| Barrett (2019) | Y | Y | Y | Y | Y | Y | Y | Y | N | Y | Y | Y | Y | Y | N | N | Y | N | N | Y | N | Y | N | Y | Y | Y | Y | Y | Y | N | Y | Y | Y | N | N | — | — | — | N | N | N | N | Y | N |
| Beck (2023) | N | Y | N | Y | Y | Y | Y | Y | Y | Y | Y | Y | Y | Y | N | N | Y | N | N | Y | N | Y | N | Y | N | Y | N | N | Y | Y | Y | Y | Y | Y | N | — | — | — | N | N | N | N | Y | N |
| Bouck (2018) | N | Y | Y | Y | Y | Y | N | N | N | Y | Y | Y | Y | Y | N | N | Y | N | N | Y | N | N | N | Y | N | Y | N | N | Y | N | Y | Y | Y | Y | N | — | — | — | N | N | N | N | N | N |
| Bowman-Perrott (2016) | N | Y | N | Y | N | N | Y | Y | Y | Y | Y | Y | Y | Y | N | Y | N | N | N | Y | N | N | N | N | N | Y | N | N | N | N | N | N | N | Y | N | — | — | — | Y | N | N | N | N | N |
| Boyle (2017) | N | Y | N | Y | Y | Y | Y | N | N | Y | Y | Y | Y | Y | N | N | N | N | N | Y | N | N | N | N | N | Y | N | N | N | N | N | N | N | N | N | — | — | — | N | N | N | N | Y | N |
| Brodsky (2018) | N | Y | Y | Y | N | N | Y | N | N | Y | N | Y | Y | Y | N | N | Y | N | N | Y | N | N | N | N | N | Y | N | N | N | N | N | N | N | Y | N | N | Y | Y | Y | N | N | N | Y | N |
| Brown (2018) | N | Y | N | N | N | N | Y | Y | Y | Y | Y | Y | Y | Y | N | Y | Y | N | N | Y | N | Y | N | Y | N | Y | N | N | N | N | N | N | N | Y | N | — | — | — | N | N | N | N | N | N |
| Cameron (2001) | N | Y | N | N | N | N | Y | Y | Y | N | N | Y | N | Y | N | Y | N | N | N | Y | N | N | N | N | N | Y | N | N | N | N | N | N | N | Y | Y | Y | Y | Y | N | N | N | Y | N | N |
| Carvalho (2022) | N | N | N | N | N | N | N | N | N | N | Y | Y | Y | N | N | N | N | N | N | Y | N | Y | Y | Y | N | Y | N | N | N | N | N | N | N | Y | N | — | — | — | N | N | N | Y | Y | N |
| Contreras (2023) | N | N | N | Y | Y | Y | Y | Y | Y | Y | Y | Y | Y | Y | N | N | Y | N | N | Y | N | Y | N | Y | N | Y | N | N | N | N | N | N | N | N | N | — | — | — | Y | N | N | N | Y | N |
| DeSouza (2017) | N | Y | N | N | N | N | Y | N | N | Y | Y | Y | Y | N | Y | N | N | N | N | N | N | N | N | N | N | N | N | N | N | N | N | N | N | N | N | — | — | — | N | N | N | N | Y | N |
| Dowdy (2020) | N | Y | N | Y | Y | Y | N | N | N | Y | N | Y | Y | Y | N | Y | Y | N | N | Y | N | N | N | Y | Y | Y | N | Y | Y | Y | Y | Y | Y | Y | N | N | N | N | Y | N | Y | N | N | N |
| Dowdy (2022) | N | N | N | Y | N | N | Y | Y | Y | N | Y | Y | N | N | N | N | N | N | N | N | N | N | N | N | N | N | N | N | N | N | N | N | N | Y | N | — | — | — | N | N | N | N | Y | N |
| Dunn (2017) | N | Y | N | Y | N | N | Y | Y | Y | Y | Y | Y | Y | Y | Y | N | Y | N | N | Y | N | N | N | Y | Y | Y | N | Y | Y | Y | N | Y | N | Y | N | N | N | N | Y | N | N | Y | N | N |
| Ennis (2017) | N | Y | N | Y | N | Y | Y | Y | Y | Y | N | Y | Y | Y | Y | N | Y | N | N | Y | N | N | N | Y | N | Y | N | Y | Y | Y | Y | Y | Y | Y | N | — | — | — | Y | N | N | N | N | N |
| Erion (2006) | N | Y | N | N | N | N | Y | Y | Y | Y | Y | Y | Y | Y | N | Y | Y | N | N | Y | N | N | N | Y | N | N | N | N | N | N | N | N | N | Y | N | Y | Y | Y | Y | N | N | N | N | N |
| Fox (2021) | N | Y | N | Y | Y | Y | Y | Y | Y | Y | Y | Y | Y | Y | N | N | Y | N | N | Y | N | Y | N | Y | N | Y | N | N | N | N | N | N | N | Y | N | — | — | — | Y | N | N | Y | N | N |
| Frampton (2021) | N | N | N | Y | N | Y | Y | Y | Y | Y | N | Y | Y | N | N | N | Y | N | N | Y | N | N | N | Y | Y | Y | N | N | Y | Y | Y | Y | Y | Y | N | — | — | — | N | N | N | N | N | N |
| Gardner (2012) | N | Y | Y | N | N | N | N | N | N | Y | Y | Y | Y | N | Y | N | Y | N | N | Y | N | N | N | Y | Y | Y | N | Y | Y | Y | Y | Y | Y | Y | N | — | — | — | N | N | N | N | N | N |
| Germansky (2022) | N | Y | Y | Y | Y | Y | Y | N | N | Y | N | Y | Y | Y | N | N | Y | N | N | Y | N | Y | N | Y | Y | Y | N | N | Y | Y | N | Y | Y | Y | N | — | — | — | N | N | N | Y | Y | N |
| Groves (2023) | Y | Y | Y | Y | Y | Y | Y | Y | Y | Y | N | Y | Y | Y | N | N | Y | N | N | Y | N | Y | N | Y | Y | Y | Y | N | Y | Y | Y | Y | Y | Y | N | — | — | — | N | N | N | N | Y | N |
| Haddock (2020) | N | Y | N | Y | N | Y | N | Y | Y | Y | Y | Y | Y | Y | N | Y | Y | N | Y | Y | N | N | N | Y | Y | Y | N | N | N | N | N | N | N | N | N | N | N | N | Y | N | N | Y | N | N |
| Hawken (2014) | N | Y | N | N | N | N | Y | Y | Y | Y | Y | Y | Y | Y | N | Y | Y | N | N | Y | N | N | N | Y | N | Y | N | N | N | N | N | N | N | Y | N | — | — | — | N | N | N | N | N | N |
| Heinicke (2019) | N | Y | N | N | N | N | Y | Y | Y | Y | Y | Y | Y | Y | Y | N | Y | N | N | Y | N | Y | N | Y | Y | Y | N | N | N | N | N | N | N | Y | N | — | — | — | N | N | N | N | N | N |
| Hirsch (2021) | N | Y | N | Y | N | Y | Y | Y | Y | N | N | Y | Y | Y | Y | N | Y | N | N | Y | N | Y | N | Y | Y | Y | N | N | Y | Y | Y | N | N | Y | N | — | — | — | N | N | N | N | Y | N |
| Hurd (2023) | N | Y | N | Y | Y | Y | Y | Y | Y | Y | Y | Y | Y | Y | N | N | Y | N | N | Y | N | N | N | Y | Y | Y | N | N | N | N | N | N | N | Y | N | — | — | — | N | N | N | N | Y | N |
| Jaehnig (2007) | N | N | N | N | N | N | N | N | N | Y | N | Y | Y | N | N | N | Y | N | N | Y | N | N | N | N | N | N | N | N | N | N | N | N | N | N | N | — | — | — | N | N | N | N | N | N |
| Kestner (2023) | N | Y | N | Y | N | N | Y | Y | Y | Y | Y | Y | Y | Y | N | N | Y | N | N | Y | N | N | N | Y | Y | Y | N | N | N | N | N | N | N | Y | N | — | — | — | Y | N | N | N | Y | N |
| King (2019) | N | Y | N | Y | Y | Y | Y | Y | Y | Y | Y | Y | Y | Y | Y | Y | Y | N | N | Y | N | N | N | Y | N | Y | N | N | Y | Y | N | Y | Y | Y | Y | — | — | — | N | N | N | N | N | N |
| Konrad (2009) | N | Y | N | Y | N | Y | Y | N | Y | Y | Y | Y | Y | Y | Y | N | Y | N | N | Y | N | N | N | Y | Y | Y | N | N | N | N | N | N | N | Y | N | N | N | N | N | N | N | N | N | N |
| Kranak (2023) | N | Y | N | Y | Y | Y | Y | Y | Y | Y | Y | Y | Y | Y | Y | Y | Y | N | N | Y | N | Y | N | Y | N | Y | N | N | N | N | N | N | N | N | N | — | — | — | N | N | N | N | Y | N |
| Kupzyk (2023) | N | Y | N | N | N | N | Y | Y | Y | N | Y | Y | Y | Y | N | N | Y | N | N | Y | N | Y | N | Y | Y | Y | N | N | Y | Y | Y | Y | Y | Y | N | — | — | — | Y | N | N | Y | Y | N |
| Losinski (2017) | N | Y | Y | Y | Y | Y | N | N | N | Y | N | Y | Y | Y | Y | Y | N | N | N | Y | N | N | N | Y | N | Y | N | N | Y | Y | Y | Y | Y | N | N | N | Y | N | Y | N | Y | N | N | N |
| MacSuga-Gage (2015) | N | Y | Y | Y | N | N | Y | N | N | Y | Y | Y | N | Y | N | N | Y | N | N | Y | N | Y | N | Y | N | Y | N | N | N | N | N | N | N | Y | N | — | — | — | N | N | N | Y | N | N |
| Maggin (2014) | N | Y | Y | Y | Y | Y | Y | Y | Y | Y | Y | Y | Y | Y | N | Y | Y | N | N | Y | N | Y | N | Y | N | Y | N | N | Y | Y | Y | Y | Y | Y | Y | Y | Y | N | N | N | N | N | N | N |
| McCormack (2019) | N | Y | N | Y | Y | Y | Y | Y | Y | Y | Y | Y | Y | N | N | Y | N | N | N | Y | N | Y | N | Y | N | Y | N | N | Y | Y | Y | Y | N | Y | Y | Y | Y | Y | Y | N | Y | N | N | N |
| McCoy (2019) | N | Y | N | N | N | N | N | N | N | Y | N | Y | Y | Y | N | N | Y | N | N | Y | N | N | N | N | N | Y | N | Y | Y | Y | Y | Y | Y | N | N | — | — | — | N | N | N | Y | Y | N |
| Nemer (2019) | N | Y | N | N | N | N | Y | N | Y | Y | Y | Y | Y | Y | Y | N | Y | N | N | Y | N | Y | N | Y | N | Y | N | N | N | N | N | N | N | Y | N | — | — | — | Y | N | N | Y | N | N |
| Nesselrode (2022) | N | Y | N | N | N | N | Y | N | N | Y | N | Y | Y | Y | Y | N | N | N | Y | Y | N | Y | N | Y | N | Y | N | N | N | N | N | N | N | N | N | — | — | — | N | N | N | Y | Y | N |
| Odum (2020) | N | Y | N | N | N | N | N | N | N | N | N | Y | Y | N | N | N | N | N | N | Y | N | N | N | Y | N | Y | N | N | N | N | N | N | N | N | Y | N | N | N | Y | N | Y | N | N | N |
| Page (2020) | N | N | N | Y | N | N | Y | N | N | Y | Y | Y | Y | N | Y | N | Y | N | N | Y | N | N | N | Y | Y | Y | N | N | N | N | N | N | N | N | N | — | — | — | Y | N | N | N | Y | N |
| Park (2020) | N | Y | N | N | N | N | Y | Y | Y | Y | Y | Y | Y | Y | N | N | N | N | N | Y | N | Y | N | Y | Y | Y | N | N | Y | Y | N | Y | Y | Y | Y | Y | Y | Y | Y | Y | Y | Y | Y | N |
| Perrin (2022) | N | Y | N | Y | N | Y | Y | Y | Y | N | Y | Y | Y | N | N | N | Y | N | Y | Y | N | N | N | Y | Y | Y | N | N | N | N | N | N | N | Y | Y | — | — | — | N | N | N | N | Y | N |
| Podlesnik (2023) | N | N | N | Y | Y | Y | Y | Y | Y | Y | N | Y | Y | Y | Y | N | Y | N | N | Y | N | N | N | Y | N | Y | N | N | N | N | N | N | N | Y | N | — | — | — | N | N | N | N | Y | N |
| Rajaraman (2020) | N | Y | N | N | N | N | Y | N | Y | Y | Y | Y | Y | Y | N | N | Y | N | N | Y | N | Y | N | Y | Y | Y | N | N | N | N | N | N | N | Y | N | — | — | — | N | N | N | N | N | N |
| Regnier (2022) | N | N | N | Y | Y | Y | N | N | N | Y | N | Y | Y | Y | N | N | Y | N | N | Y | N | Y | N | Y | N | Y | N | N | N | N | N | N | N | Y | N | — | — | — | N | N | N | Y | N | N |
| Richman (2015) | N | Y | N | Y | Y | Y | Y | Y | Y | Y | Y | Y | Y | Y | N | N | Y | N | N | Y | N | N | N | Y | Y | Y | N | N | N | N | N | N | N | N | Y | N | Y | Y | Y | N | N | N | N | N |
| Riden (2022) | N | N | N | Y | Y | Y | Y | Y | Y | Y | N | Y | Y | Y | Y | Y | Y | N | N | Y | N | Y | N | Y | Y | Y | N | N | N | N | N | N | N | Y | N | — | — | — | N | N | N | N | Y | N |
| Rubio (2021) | N | N | N | Y | N | Y | Y | Y | Y | Y | Y | Y | Y | Y | N | N | Y | N | N | Y | N | Y | N | Y | Y | Y | N | Y | Y | Y | Y | Y | Y | Y | N | — | — | — | Y | N | N | N | N | N |
| Saini (2019) | N | Y | N | Y | N | Y | Y | Y | Y | Y | Y | Y | Y | Y | N | N | Y | N | N | Y | N | Y | N | Y | N | Y | N | Y | Y | Y | Y | N | N | N | N | — | — | — | N | N | N | N | N | N |
| Saini (2020) | N | Y | N | Y | N | Y | Y | Y | Y | Y | Y | Y | Y | Y | N | N | Y | N | N | Y | N | Y | N | Y | Y | Y | N | N | N | N | N | N | N | N | N | — | — | — | N | N | N | N | N | N |
| Sivaraman (2020) | N | Y | N | Y | Y | Y | Y | Y | Y | Y | Y | Y | Y | Y | N | Y | Y | N | N | Y | N | Y | N | Y | Y | Y | N | N | Y | Y | Y | Y | Y | N | N | — | — | — | N | N | N | N | N | N |
| Sivaraman (2023) | N | N | N | Y | N | Y | Y | N | Y | Y | Y | Y | Y | Y | N | N | Y | Y | Y | Y | N | Y | N | N | N | Y | N | N | N | N | N | N | N | N | N | — | — | — | N | N | N | N | Y | N |
| Stinson (2022) | N | N | N | Y | N | Y | Y | N | N | Y | Y | Y | Y | Y | N | Y | Y | N | Y | Y | N | Y | N | Y | N | Y | N | N | N | N | N | N | N | Y | N | — | — | — | N | N | N | N | Y | N |
| Suarez (2022) | N | Y | N | Y | N | N | Y | N | N | N | Y | Y | Y | Y | N | N | Y | N | N | Y | N | N | N | Y | N | Y | N | N | N | N | N | N | N | Y | N | — | — | — | N | N | N | N | Y | N |
| Sweigart (2016) | N | Y | N | N | Y | Y | N | Y | Y | Y | Y | Y | Y | Y | N | N | Y | N | N | Y | N | N | N | Y | N | Y | N | N | Y | Y | Y | Y | Y | Y | N | — | — | — | N | N | N | N | N | N |
| Thoele (2023) | N | Y | N | Y | Y | Y | Y | N | Y | Y | Y | Y | Y | N | Y | N | Y | N | Y | Y | N | N | N | Y | N | Y | N | N | N | N | N | N | N | Y | N | — | — | — | Y | N | N | Y | Y | N |
| Tincani (2020) | N | N | N | Y | N | Y | Y | Y | Y | Y | Y | Y | Y | N | Y | N | N | N | Y | Y | N | N | N | N | N | Y | N | N | N | N | N | N | N | N | N | — | — | — | N | N | N | N | N | N |
| Trevor (2021) | N | N | N | N | N | N | Y | Y | Y | Y | Y | Y | Y | Y | N | N | Y | N | N | Y | Y | Y | Y | Y | Y | Y | N | N | Y | Y | Y | Y | Y | Y | N | N | N | N | Y | N | N | Y | Y | N |
| Weinsztok (2023) | N | Y | Y | Y | Y | Y | Y | Y | Y | Y | Y | Y | Y | Y | N | Y | N | N | Y | Y | N | Y | N | Y | Y | Y | N | N | N | N | N | N | N | Y | N | — | — | — | Y | N | N | N | Y | N |
| White (1988) | N | N | N | N | N | N | N | N | N | N | N | Y | Y | Y | N | N | N | N | N | Y | N | N | N | N | N | Y | N | N | N | N | N | N | N | N | N | N | N | N | N | N | N | N | N | N |
| Wong (2023) | N | Y | N | Y | N | Y | Y | N | Y | Y | Y | Y | Y | Y | Y | Y | Y | N | N | Y | N | Y | N | Y | Y | Y | N | N | Y | N | Y | N | N | N | Y | — | — | — | Y | N | N | N | Y | N |
| Wooderson (2022) | N | N | Y | Y | N | Y | N | N | N | Y | Y | Y | Y | Y | N | N | Y | N | N | Y | N | N | N | Y | Y | Y | N | N | Y | Y | Y | N | N | N | Y | N | N | N | Y | N | N | Y | Y | N |
| Note. Y = criterion present; N = criterion absent; — = not applicable | | | | | | | | | | | | | | | | | | | | | | | | | | | | | | | | | | | | | | | | | | | | |
| R-AMSTAR items are shown at the sub-item level (e.g., 1a–1c, 2a–2f). | | | | | | | | | | | | | | | | | | | | | | | | | | | | | | | | | | | | | | | | | | | | |
