# Supplementary material for: Evaluating the quality of systematic reviews and meta-analyses published in behaviour analysis journals: An umbrella review
Source: PLoS One. 2026 Jun 26;21(6):e0350142. doi: 10.1371/journal.pone.0350142 (PMC13309035; doi:10.1371/journal.pone.0350142)
Supplement: S8 File — (DOCX) [file pone.0350142.s008.docx]

Full search terms

Searches were conducted in APA PsycINFO via the ProQuest interface.

| Publication | Search String |
| --- | --- |
| Education and Treatment of Children | publication("education and treatment of children") AND tiab("meta-analy*" OR "systematic review") |
| Analysis of Verbal Behavior | publication("analysis of verbal behavior") AND tiab("meta-analy*" OR "systematic review") |
| Journal of Applied Behavior Analysis | publication("journal of applied behavior analysis") AND tiab("meta-analy*" OR "systematic review") |
| Perspectives on Behavior Science | publication("perspectives on behavior science") AND tiab("meta-analy*" OR "systematic review") |
| The Behavior Analyst | publication("the behavior analyst") AND tiab("meta-analy*" OR "systematic review") |
| The Psychological Record | publication("the psychological record") AND tiab("meta-analy*" OR "systematic review") |
| Behavior Analysis in Practice | publication("behavior analysis in practice") AND tiab("meta-analy*" OR "systematic review") |
| Journal of the Experimental Analysis of Behavior | publication("journal of the experimental analysis of behavior") AND tiab("meta-analy*" OR "systematic review") |
